# The Role of Prior-Induced Regularization in Accuracy and Stability of Genomic Prediction Across Unimodal and Multimodal Models

**DOI:** 10.3390/plants15162430

**Published:** 2026-08-10

**Authors:** Osval A. Montesinos-López, José Elías Peregrina-Chavarría, Abelardo Montesinos-López, José Crossa, Ivana N. Briseño-Rodríguez, Roberto de la Rosa Santa-María, Nereyda C. Pérez-González, Karol D. Johnston-Navarro, Mayte Muñoz-Rosales, Verónica M. Guzmán-Sandoval, Iván Delgado-Enciso, Luis Posadas, Reka Howard

**Affiliations:** 1 Facultad de Telemática, Universidad de Colima, Colima 28040, Mexico; osval78t@gmail.com (O.A.M.-L.); jperegrina4@ucol.mx (J.E.P.-C.); ibriseno0@ucol.mx (I.N.B.-R.); nperez17@ucol.mx (N.C.P.-G.); kjohnston@ucol.mx (K.D.J.-N.); mmunoz14@ucol.mx (M.M.-R.); 2 Centro Universitario de Ciencias Exactas e Ingenierías (CUCEI), Universidad de Guadalajara, Guadalajara 44430, Mexico; 3 Post-Graduate College (COLPOS) Montecillos, Montecillos 56230, Mexico; j.crossa@outlook.com; 4 Producción Agroalimentaria en el Trópico, Colegio de Postgraduados (CP), Campus Tabasco, H. Cárdenas 86500, Mexico; robdelarosas@colpos.mx; 5 Facultad de Psicología, Universidad de Colima, Colima 28040, Mexico; gus_vero@ucol.mx; 6 School of Medicine, University of Colima, Colima 28040, Mexico; ivan_delgado_enciso@ucol.mx; 7 Department of Agronomy and Horticulture, University of Nebraska-Lincoln, Lincoln, NE 68583, USA; lposadas2@huskers.unl.edu; 8 Department of Statistics, University of Nebraska-Lincoln, Lincoln, NE 68583, USA

**Keywords:** Bayesian genomic prediction, prior-induced regularization, Bayesian shrinkage, genomic selection, multimodal prediction, genotype-by-environment interaction, wheat, rice, potato

## Abstract

In this study, we assessed the impact of prior-induced regularization using six real datasets from wheat, rice, and potato, spanning 107–758 genotypes, 2–12 environments, 1–18 traits, and 2744–108,024 molecular markers. Two modeling scenarios were evaluated: (i) unimodal genomic prediction based solely on marker information and (ii) multimodal (multi-component) prediction integrating genomic, environmental, and genotype-by-environment (G × E) effects. Predictive performance was evaluated using Pearson’s correlation (COR) and normalized root mean squared error (NRMSE) under 10 repeated random 50% training–50% testing partitions, representing prediction of untested lines in tested environments. Bayesian genomic prediction (BGP) relies on prior distributions to regulate shrinkage and stabilize inference in high-dimensional settings. We evaluated whether predictive performance was driven primarily by the type of Bayesian prior or by the presence of effective prior-induced regularization. Across most datasets, regularized Bayesian models achieved higher predictive correlations and markedly lower NRMSE than the weakly regularized or unregularized baseline. Differences among regularized prior families were generally modest, whereas weakening or removing regularization frequently produced unstable estimates and inflated prediction error. Predictive results were obtained for both winter-wheat datasets as well as for the rice, potato, and DMario datasets. In multimodal analyses, models with coherent regularization across genomic, environmental, and genotype-by-environment components were generally more accurate and stable than configurations in which regularization was absent or weakened in key components. Rice_Kim_2020 was an informative exception in which the baseline remained competitive. These results show that the principal empirical contrast is the presence versus absence of effective prior-induced regularization, rather than a universal ranking of Bayesian prior families. Appropriate regularization should therefore be treated as a central model-design decision in genomic prediction.

## 1. Introduction

Genomic selection (GS) has fundamentally transformed modern plant and animal breeding by enabling the prediction of genetic merit using genome-wide molecular markers, often before complete phenotypic information is available [1,2,3]. This shift from phenotype-based to genome-enabled selection has accelerated genetic gain in major crops such as wheat, rice, and potato, as well as in livestock systems, particularly for complex traits that are costly, time-consuming, or environmentally dependent to measure [4,5]. Despite these advances, achieving high predictive accuracy in real breeding programs remains challenging because genomic prediction datasets are commonly characterized by limited sample size, unbalanced experimental designs, environmental heterogeneity, and highly polygenic trait architectures [6,7].

A defining feature of genomic prediction is its high-dimensional structure, where the number of molecular markers (*p*) typically exceeds, and often greatly exceeds, the number of phenotyped individuals (*n*). In such settings (*p* ≫ *n*), statistical estimation is intrinsically ill-posed, and models without adequate regularization may produce unstable estimates, inflated error variance, and poor generalization. Bayesian genomic prediction (BGP) models provide a flexible framework for addressing this problem because prior distributions regulate the magnitude and dispersion of marker effects, stabilize estimation, and reduce overfitting [8,9]. Therefore, in this study, prior specification is interpreted primarily through its role as a regularization mechanism. The central empirical comparison is not intended as a strict philosophical contrast between informative and non-informative Bayesian priors, but rather as an evaluation of how prior-induced regularization affects predictive accuracy and stability in high-dimensional genomic prediction.

A wide range of BGP models has been proposed, often referred to as the “Bayesian alphabet,” including Bayesian Ridge Regression (BRR), Bayes A, Bayes B, Bayes C, and the Bayesian Lasso [10,11,12]. These models differ in how they impose shrinkage on marker effects. BRR assumes homogeneous shrinkage across all markers and is commonly appropriate for highly polygenic traits, whereas Bayes A and Bayes B allow marker-specific variance heterogeneity and can accommodate genetic architectures involving both small and large effects. Bayes C incorporates variable selection, while the Bayesian Lasso imposes shrinkage toward zero with adaptive flexibility [11,12]. Although these priors differ statistically, their practical contribution in high-dimensional genomic prediction is often expressed through their capacity to regularize marker-effect estimation.

Kernel-based models, particularly reproducing kernel Hilbert space (RKHS) regression, provide another important perspective on prior-induced regularization. Instead of assigning priors directly to individual marker effects, RKHS models place priors on functions through a kernel-defined covariance structure [12,13]. This framework can capture nonlinear relationships, epistasis, and complex genotype–phenotype patterns without explicitly specifying all interaction terms. Thus, RKHS models can also be understood as providing an implicit form of regularization, where the kernel structure controls model complexity and stabilizes prediction.

The importance of regularization becomes even more pronounced in multi-environment and multimodal (multi-component) genomic prediction models integrating genomic, environmental, and genotype-by-environment components. In these settings, phenotypic variation arises not only from genomic effects, but also from environmental factors and genotype-by-environment (G × E) interactions [14,15]. Modern prediction models increasingly integrate genomic, environmental, and interaction kernels within a unified framework. Although such models provide a more realistic representation of biological and breeding processes, they also introduce additional model complexity and multiple variance components. Consequently, effective regularization is essential for stable estimation, coherent variance allocation, and reliable prediction of (G × E) effects.

In this context, the distinction between regularized Bayesian models and weakly regularized or unregularized baseline formulations is critical. Informative Bayesian priors introduce structured shrinkage by constraining parameter estimates toward statistically and biologically plausible regions. In contrast, weakly regularized or unregularized fixed-effect baseline formulations provide little control over parameter dispersion and may lead to unstable solutions, particularly when (*p* ≫ *n*). Thus, throughout this study, the term “prior effect” should be understood mainly as the regularization induced by the prior structure, rather than as a fine-scale ranking of alternative Bayesian prior families.

**Figure A1:**
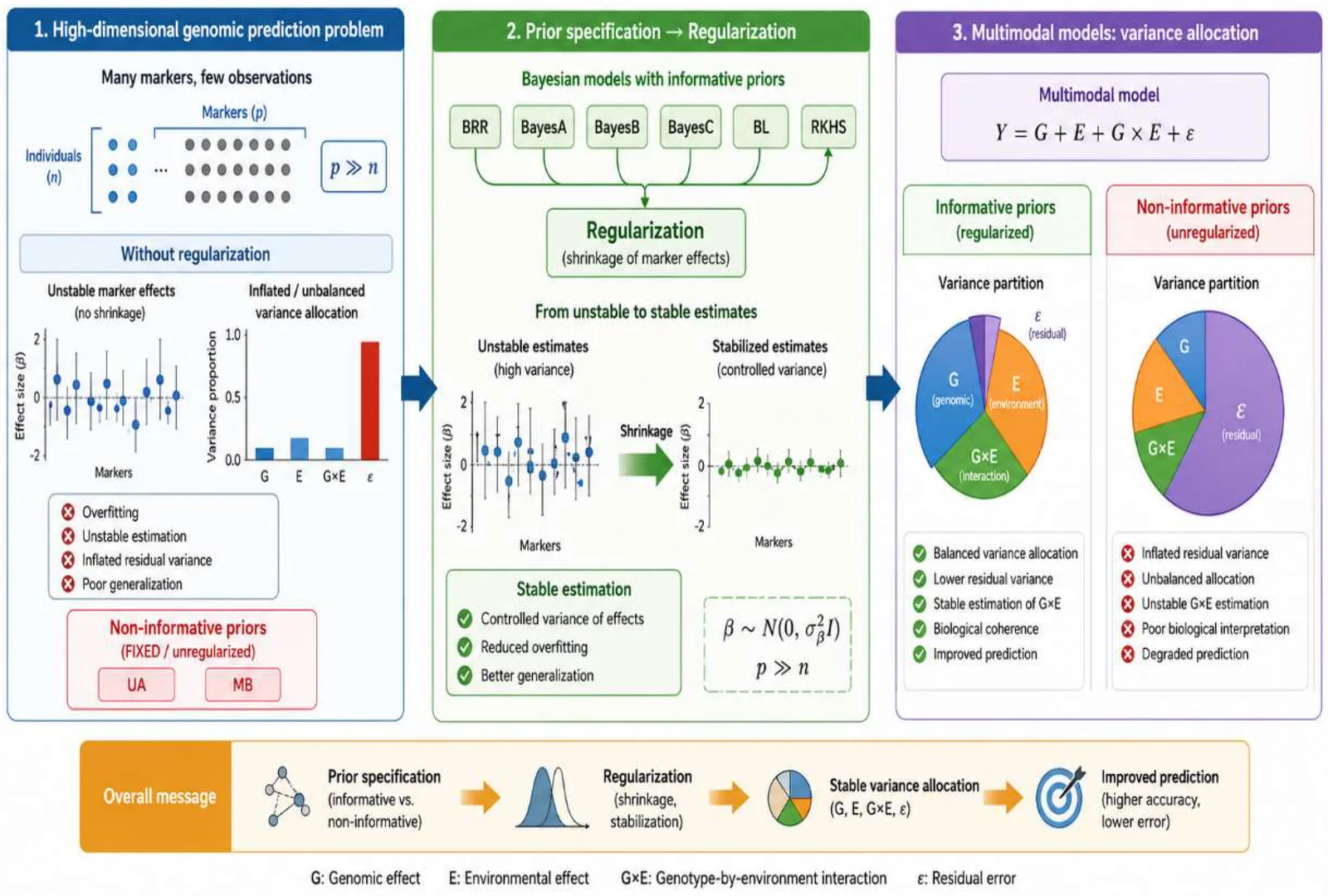
Prior specification as a regularization mechanism in genomic prediction and multimodal modeling. Colors are used to distinguish the main conceptual components of the framework: blue denotes the high-dimensional genomic-prediction problem and genomic effects; green denotes informative-prior/regularized components and stabilized estimation; purple denotes the multimodal modeling framework and residual variance; and red denotes weakly regularized or unregularized components and associated undesirable outcomes. In the variance-partition diagrams, blue represents the genomic effect (G), orange the environmental effect (E), green the genotype-by-environment interaction (G × E), and purple the residual error (ε).

G×E The conceptual framework underlying this study is summarized in Figure A1. Panel 1 illustrates the consequences of weakly regularized or unregularized estimation in high-dimensional genomic prediction, including unstable marker-effect estimates, inflated residual variance, overfitting, and poor generalization. Panel 2 shows how regularized Bayesian priors, including BRR, Bayes A, Bayes B, Bayes C, Bayesian Lasso, and RKHS-based formulations, stabilize estimation through shrinkage and control of marker-effect variance. Panel 3 extends this principle to multimodal (multi-component) genomic prediction models integrating genomic (G), environmental (E), and genotype-by-environment interaction (G × E) components. In this framework, prior-induced regularization contributes to more stable variance partitioning across model components, improved estimation of interaction effects, and enhanced predictive robustness.

Despite the extensive literature comparing Bayesian genomic prediction methods, the independent contribution of regularization induced by prior structures has not been systematically evaluated across different crops, datasets, and modeling frameworks. Many studies compare alternative models, kernels, or data-integration strategies, but these comparisons often confound differences in prior specification with differences in model architecture. As a result, it remains difficult to determine whether observed performance gains arise from specific prior families or from the broader stabilizing effect of regularization.

G×E The objective of this study was to evaluate the role of prior-induced regularization in Bayesian genomic prediction using six real datasets from wheat, rice and potato. We compared predictive performance under two modeling scenarios: (i) unimodal genomic prediction based solely on marker information, and (ii) multimodal genomic prediction integrating genomic, environmental, and genotype-by-environment interaction components. Rather than presenting the study as a fine-scale ranking of alternative Bayesian priors, we focus on the empirical consequences of using regularized Bayesian formulations compared with weakly regularized or unregularized baselines. Predictive performance was assessed using Pearson’s correlation (COR) and normalized root mean squared error (NRMSE) under repeated cross-validation. By quantifying differences across datasets, crops, and modeling frameworks, we aimed to determine when prior-induced regularization improves prediction accuracy, error control, and model stability in high-dimensional genomic prediction.

## 2. Results

The results are presented in two main sections: unimodal and multimodal (multi-component) prediction. The study comprises six datasets from three crop species—wheat, rice, and potato. Predictive results for the Winter_Wheat_Washington_2020 and Winter_Wheat_Washington_2021 datasets are reported in Appendix C and Appendix E, respectively, and are included in the across-dataset summaries. Given the high-dimensional nature of the datasets analyzed (*p* ≫ *n*), the results are interpreted primarily in terms of regularization. Throughout the manuscript, predictive stability refers to consistency of out-of-sample COR and NRMSE across repeated partitions; numerical stability refers to the absence of extreme or divergent predictions; and posterior convergence refers to satisfactory MCMC mixing and convergence diagnostics. These concepts are related but were evaluated and interpreted separately.

### 2.1. Unimodal Results

#### 2.1.1. DMario Family 14 Unimodal

**Figure 1:**
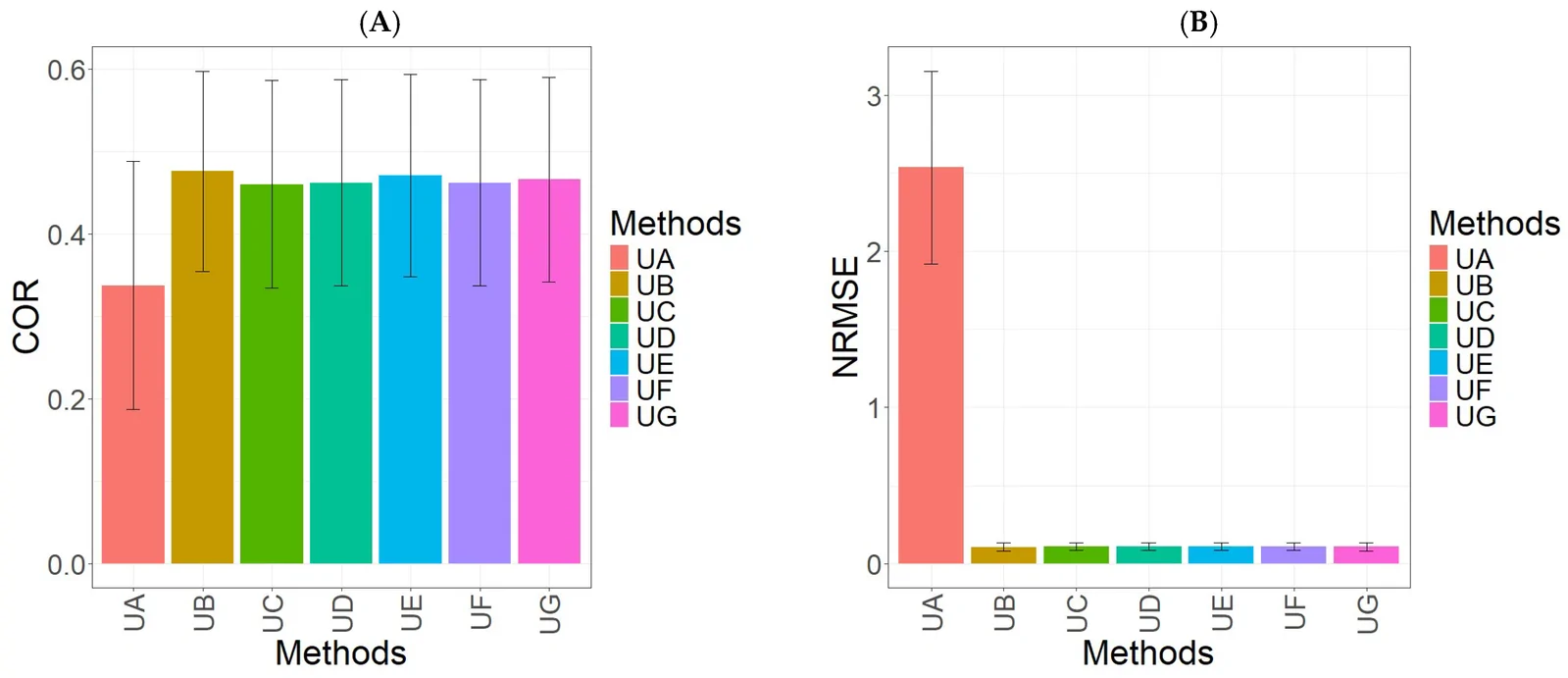
Comparative performance of prediction models for the DMario_Family14 dataset. Panel (**A**) shows results based on Pearson’s correlation (COR), and Panel (**B**) shows results based on the normalized root mean squared error (NRMSE) for the unimodal predictor. Overall, regularized Bayesian models showed more stable predictive performance than the unregularized baseline for this dataset. Error bars represent the standard error across the 10 repeated 50% training–50% testing partitions (*n* = 10 per method within each dataset/trait summary); they quantify variation among repeated data splits, not posterior uncertainty. Models are defined in Section 4.2.

Results for this dataset are shown in Figure 1 and summarized in Table A1. Model UB achieved the highest predictive performance (COR = 0.476), followed closely by Model UE (COR = 0.471), while Model UA exhibited lower performance (COR = 0.337). Differences among regularized Bayesian models (Models UB–UG) were relatively small. In terms of prediction error, most regularized models showed similar NRMSE values (approximately 0.106), whereas Model UA exhibited substantially higher error (NRMSE = 2.539). Descriptive ANOVA–Tukey summaries indicated no clear separation among models for COR, whereas NRMSE separated Model UA from the remaining models. Because the repeated cross-validation partitions are correlated, these groupings were interpreted together with effect magnitude and consistency across partitions rather than as stand-alone confirmatory inference. These results indicate that the main difference was observed between the regularized Bayesian models and the unregularized baseline model. Additional details are provided in Appendix B.

#### 2.1.2. Potato Suecia Rodomiro Ortiz 2023 Unimodal

**Figure 2:**
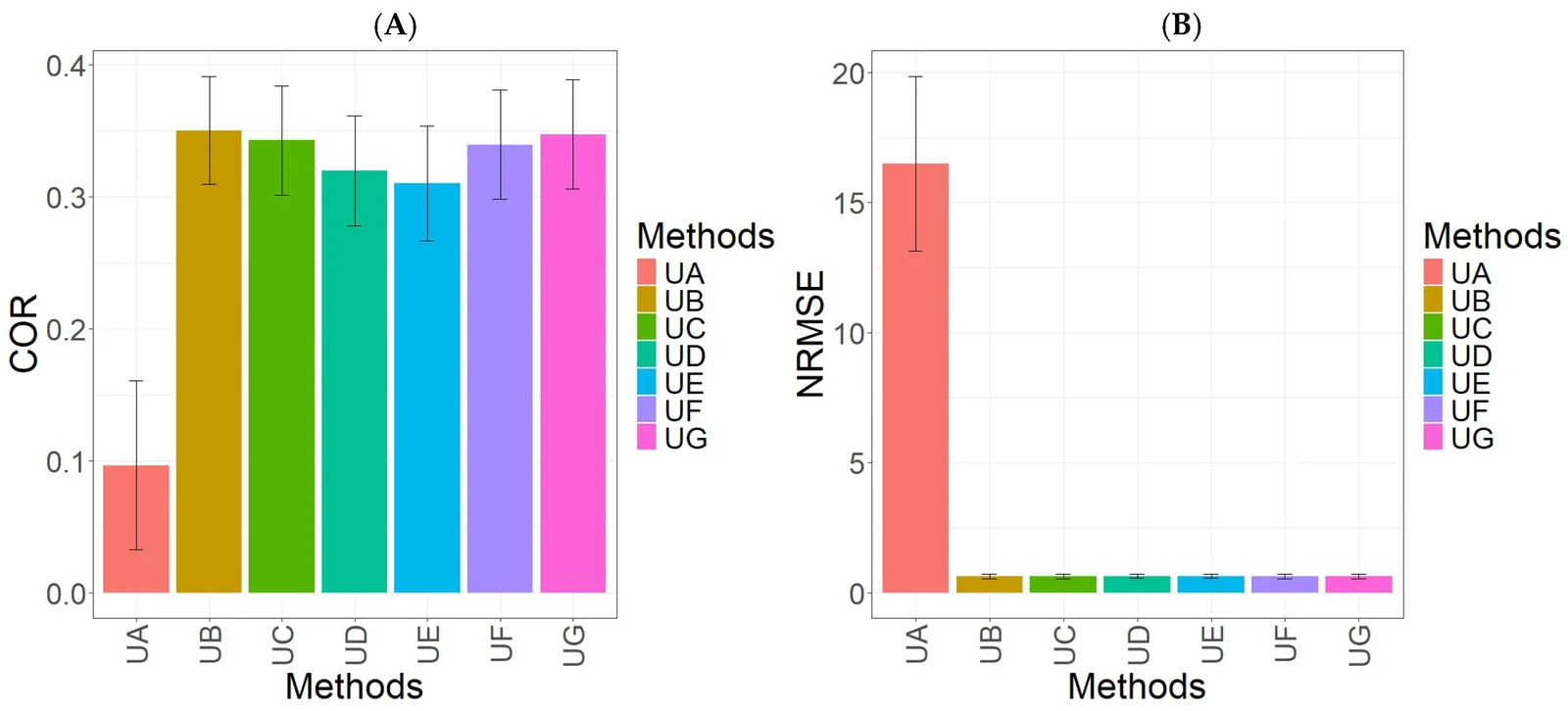
Comparative performance of prediction models for the Potato_Suecia_Rodomiro_Ortiz_2023 dataset. Panel (**A**) shows results based on Pearson’s correlation (COR), and Panel (**B**) shows results based on the normalized root mean squared error (NRMSE) for the unimodal predictor. Regularized Bayesian models outperformed the weakly regularized or unregularized baseline for this dataset. Error bars represent the standard error across the 10 repeated 50% training–50% testing partitions (*n* = 10 per method within each dataset/trait summary); they quantify variation among repeated data splits, not posterior uncertainty. Models are defined in Section 4.2.

Results are presented in Figure 2 and Table A1. Model UB achieved the highest correlation (COR = 0.350), followed closely by Model UG (COR = 0.347), whereas Model UA showed substantially lower performance (COR = 0.097). Descriptive ANOVA–Tukey summaries separated the regularized Bayesian models (UB–UG) from the weakly regularized or unregularized baseline; this separation was interpreted together with the large effect magnitude and its consistency across partitions. NRMSE values were similar across regularized models (approximately 0.626), while Model UA exhibited markedly higher error (NRMSE = 16.489). These results indicate that the observed performance differences were primarily driven by the instability of the weakly regularized or unregularized baseline, whereas regularized Bayesian models showed comparable and more stable performance. More details are provided in Appendix B.

#### 2.1.3. Rice IRRI Philippines Spindel 2015 Unimodal

**Figure 3:**
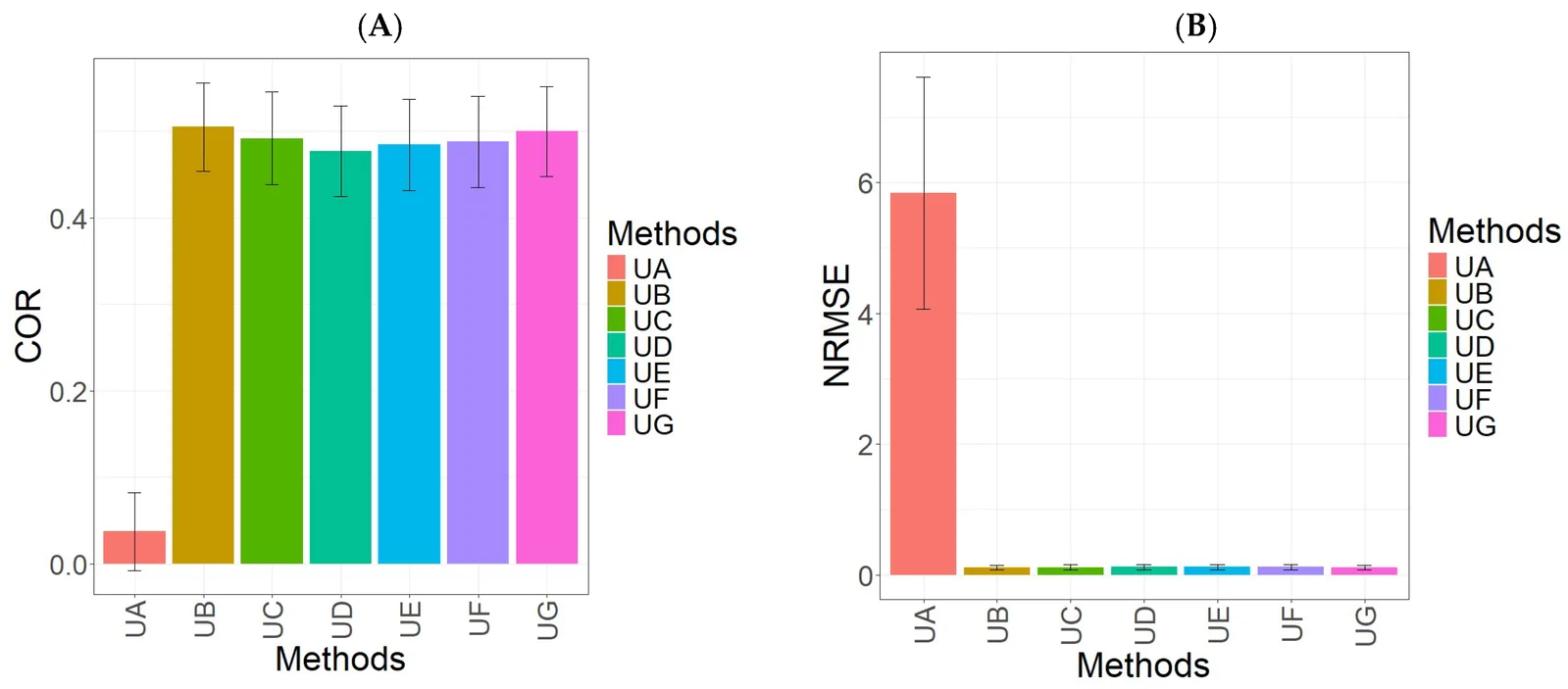
Comparative performance of prediction models for the Rice_IRRI_Philippines_Spindel_2015 dataset. Panel (**A**) shows results based on Pearson’s correlation (COR), and Panel (**B**) shows results based on the normalized root mean squared error (NRMSE) for the unimodal predictor. Regularized Bayesian models generally outperformed the weakly regularized or unregularized baseline for this dataset. Error bars represent the standard error across the 10 repeated 50% training–50% testing partitions (*n* = 10 per method within each dataset/trait summary); they quantify variation among repeated data splits, not posterior uncertainty. Models are defined in Section 4.2.

Results are shown in Figure 3 and Table A1. Model UB achieved the highest predictive accuracy (COR = 0.505), followed by Model UG (COR = 0.500), while Model UA showed very low predictive ability (COR = 0.037). Descriptive ANOVA–Tukey summaries separated the regularized Bayesian models from the weakly regularized or unregularized baseline; interpretation emphasized the large and consistent differences in COR and NRMSE rather than the grouping letters alone. In terms of NRMSE, regularized models showed consistent performance (approximately 0.115), whereas Model UA exhibited substantially higher error (NRMSE = 5.840). These results again show that the largest difference was between the regularized Bayesian models and the weakly regularized or unregularized baseline. Differences among regularized models were comparatively small. More details are provided in Appendix B.

#### 2.1.4. Rice Kim 2020 Unimodal

**Figure 4:**
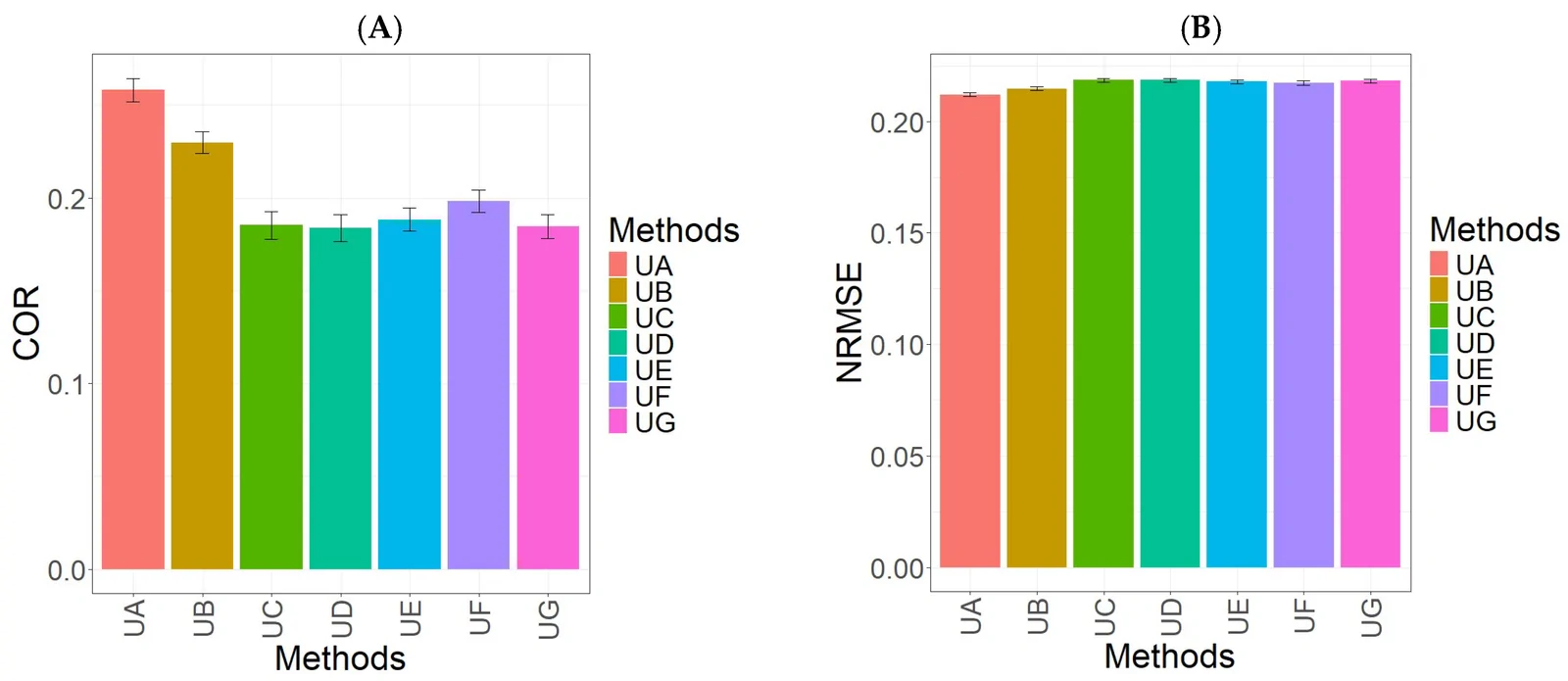
Comparative performance of prediction models for the Rice_Kim_2020 dataset. Panel (**A**) shows results based on Pearson’s correlation (COR), and Panel (**B**) shows results based on the normalized root mean squared error (NRMSE) for the unimodal predictor. In contrast to the general pattern observed in other datasets, the weakly regularized or unregularized baseline achieved the highest predictive correlation in this dataset. This exception should be interpreted cautiously and may reflect dataset-specific structure rather than a general advantage of the baseline model. Error bars represent the standard error across the 10 repeated 50% training–50% testing partitions (*n* = 10 per method within each dataset/trait summary); they quantify variation among repeated data splits, not posterior uncertainty. Models are defined in Section 4.2.

Results are presented in Figure 4 and Table A1. In contrast to the previous datasets, Model UA achieved the highest correlation (COR = 0.258), while regularized Bayesian models showed slightly lower but comparable performance (e.g., Model UB = 0.230). Descriptive ANOVA–Tukey summaries formed three groups for COR. Because the numerical differences were modest, this dataset was interpreted primarily from the observed effect sizes and the similar NRMSE values. NRMSE values were similar across models (approximately 0.212–0.218), indicating minimal differences in prediction error. This dataset represents an exception to the general pattern. Model UA achieved the highest correlation, whereas regularized Bayesian models showed slightly lower but comparable performance. The similar NRMSE values across models indicate that prediction error was not strongly affected by the regularization structure in this particular dataset. Therefore, these results suggest that, under certain data structures, simpler or weakly regularized models may perform competitively. Additional details are provided in Appendix B.

#### 2.1.5. Summary Across Datasets Unimodal

**Figure 5:**
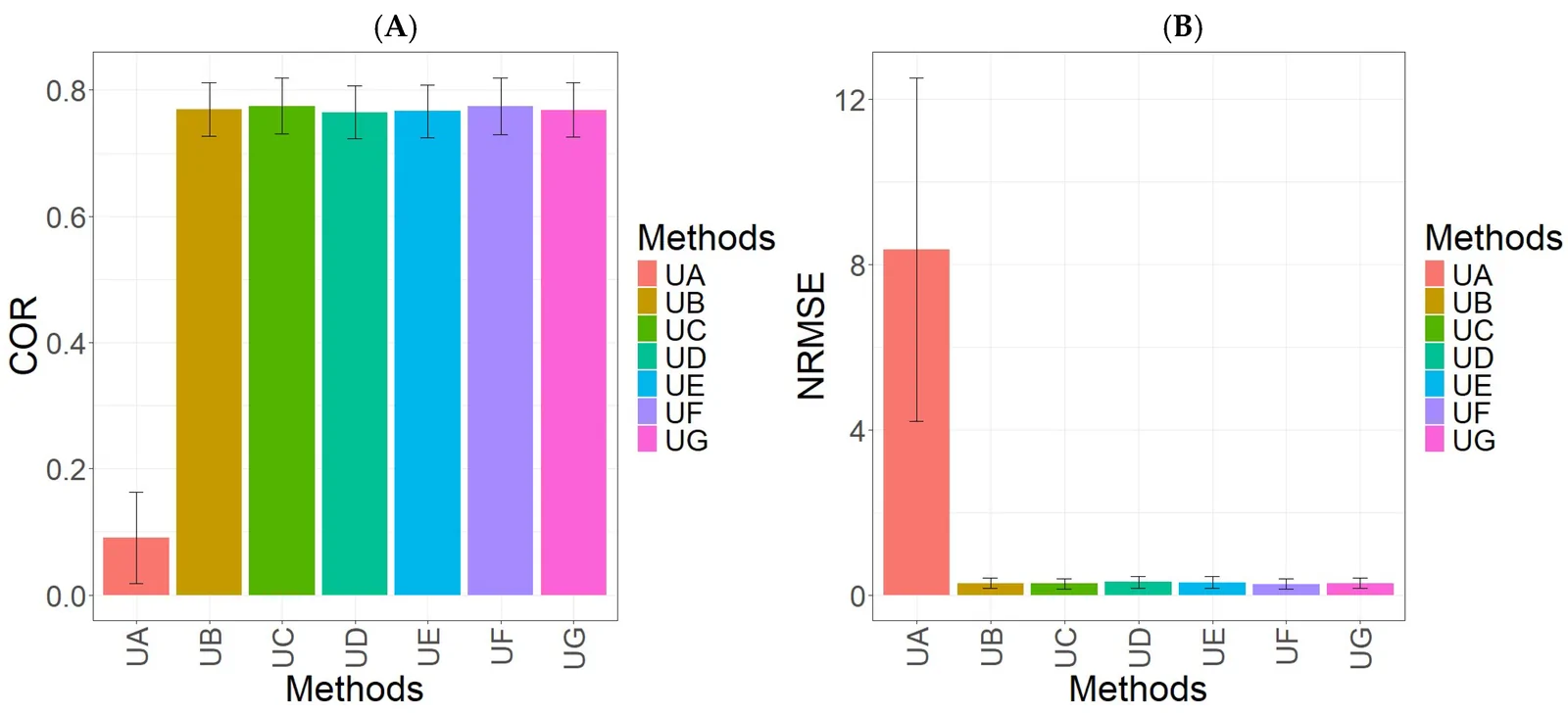
Comparative performance of prediction models for the summary across datasets. Panel (**A**) shows results based on Pearson’s correlation (COR), and Panel (**B**) shows results based on the normalized root mean squared error (NRMSE) for the unimodal input. Error bars represent the standard error across the 10 repeated 50% training–50% testing partitions (*n* = 10 per method within each dataset/trait summary); they quantify variation among repeated data splits, not posterior uncertainty. Models are defined in Section 4.2.

**Figure A2:**
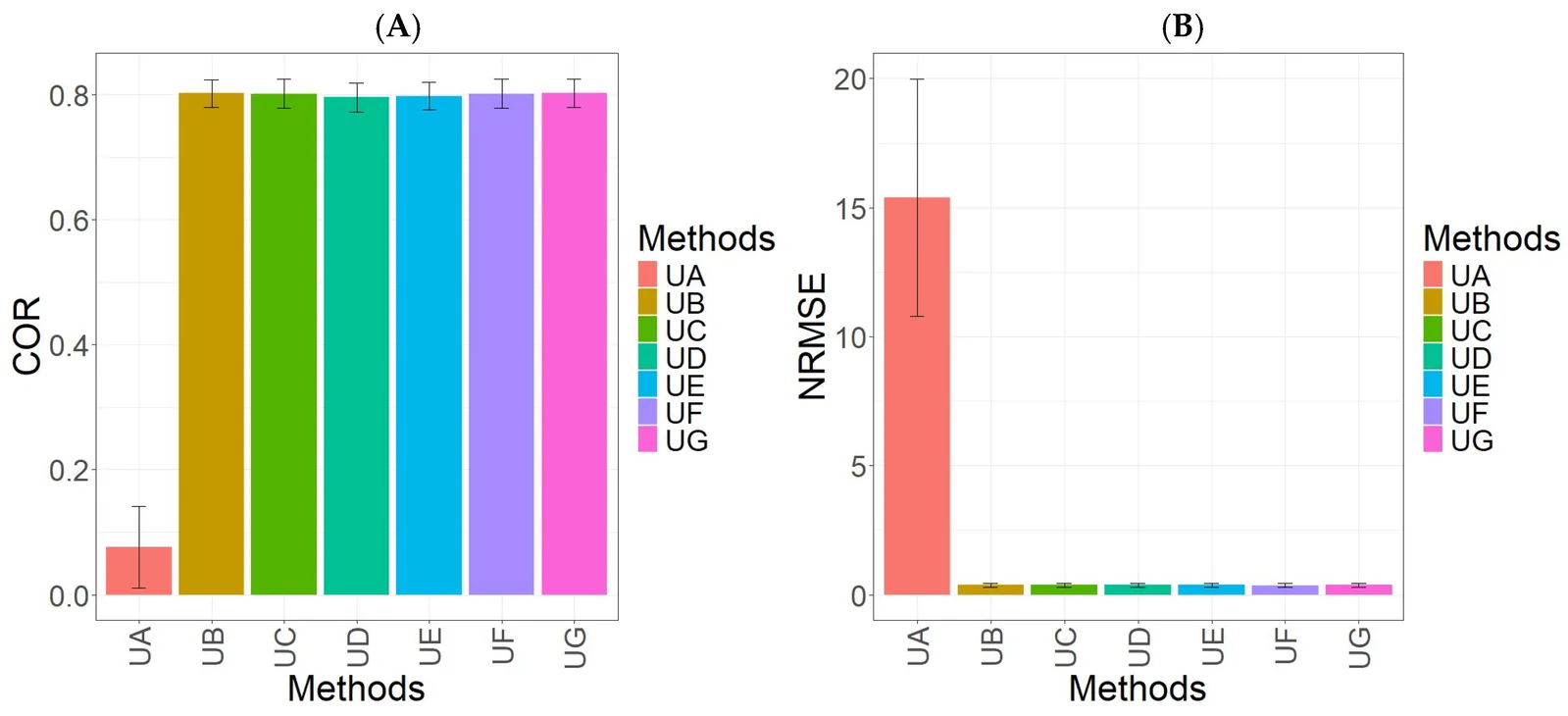
Comparative performance of prediction models for the Winter_Wheat_2020 dataset. Panel (**A**) shows results based on Pearson’s correlation (COR), and Panel (**B**) shows results based on the Normalized Root Mean Square Error (NRMSE) for the unimodal predictor. Model codes are defined in Section 4.2.

Combined results are shown in Figure 5 and Table A1. Model UB achieved the highest correlation (COR = 0.618), followed closely by Models UG and UC, whereas Model UA exhibited substantially lower performance (COR = 0.087). Descriptive ANOVA–Tukey summaries separated the regularized Bayesian models from the weakly regularized or unregularized baseline. This pattern was interpreted together with the substantial differences in predictive correlation and NRMSE. NRMSE values were similar across regularized models (approximately 0.28), while Model UA showed much higher error (NRMSE = 9.973). Across unimodal analyses, the dominant pattern was that regularized Bayesian models achieved higher predictive accuracy and lower prediction error than the weakly regularized or unregularized baseline. However, differences among regularized Bayesian models were generally modest. Therefore, the unimodal results should be interpreted primarily as evidence for the importance of regularization in high-dimensional genomic prediction, rather than as evidence that one specific Bayesian prior family is universally superior. Full details of these results are provided in Appendix B. The remaining results not shown in this section are provided in Appendix C (Table A2 and Figure A2).

### 2.2. Multimodal Results

Because the multimodal (multi-component) analysis considered a selected set of regularization configurations rather than a full factorial comparison, and because the G × E component was modeled with RKHS in all four configurations, these results identify the consequences of changing regularization in the genomic and environmental components while holding the G × E specification fixed. They do not establish that every model component independently requires the same form or degree of regularization.

#### 2.2.1. DMario Family 14 Multimodal

**Figure 6:**
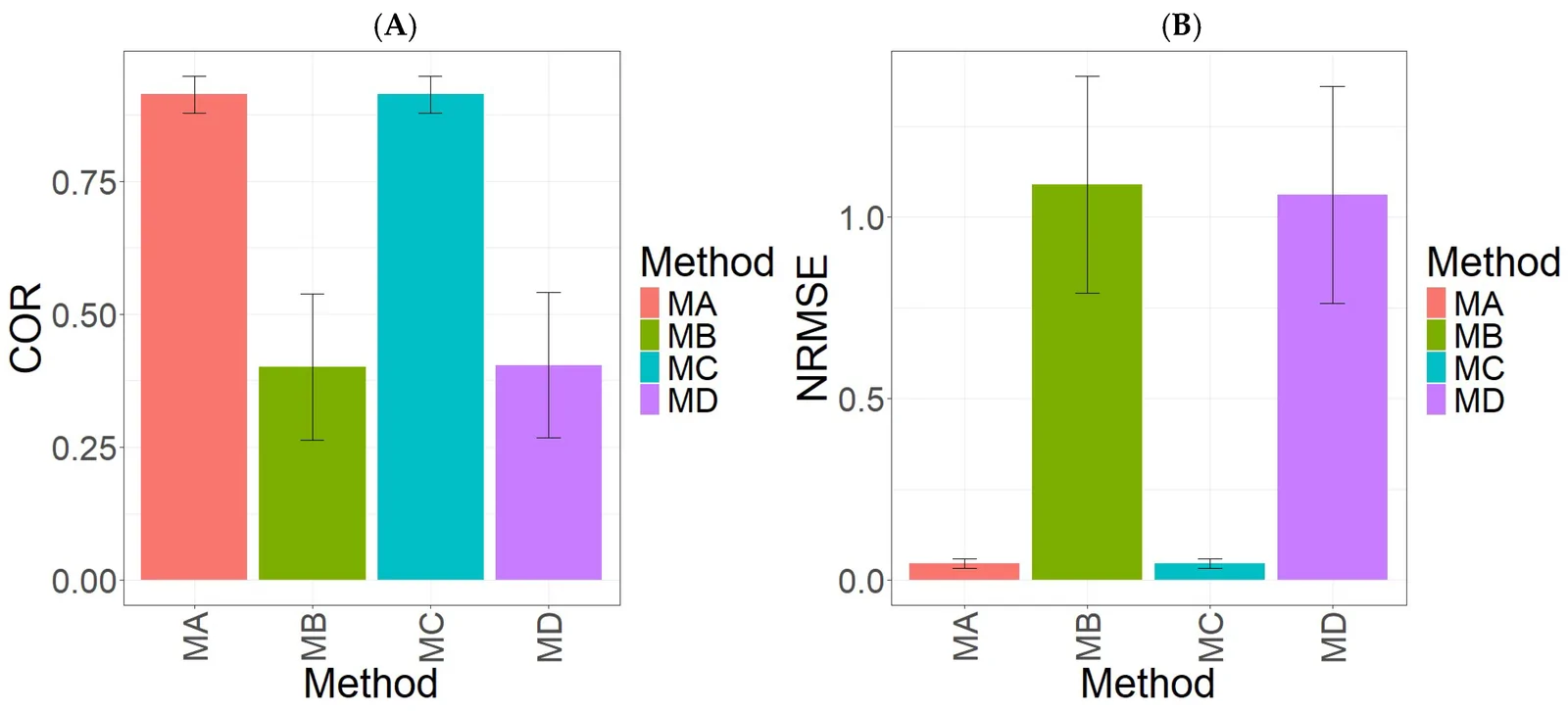
Comparative performance of prediction models for the DMario_Family14 dataset. Panel (**A**) shows results based on Pearson’s correlation (COR), and Panel (**B**) shows results based on the normalized root mean squared error (NRMSE) for the multimodal predictor. Error bars represent the standard error across the 10 repeated 50% training–50% testing partitions (*n* = 10 per method within each dataset/trait summary); they quantify variation among repeated data splits, not posterior uncertainty. Models are defined in Section 4.2.

Results are presented in Figure 6 and Table A3. Models MA and MC achieved the highest predictive performance (COR = 0.913), while Model MB showed substantially lower accuracy (COR = 0.401). Descriptive ANOVA–Tukey summaries separated these configurations. Because repeated cross-validation observations are correlated, the interpretation was based primarily on the magnitude and consistency of the predictive differences. NRMSE values followed a similar pattern, with Models MA and MC showing low error (NRMSE = 0.046) compared to Model MB (NRMSE = 1.090). The large contrast between Models MA/MC and Model MB indicates that predictive performance was strongly affected by whether effective regularization was present across model components. Additional details of these results are provided in Appendix D.

#### 2.2.2. Potato Suecia Rodomiro Ortiz 2023 Multimodal

**Figure 7:**
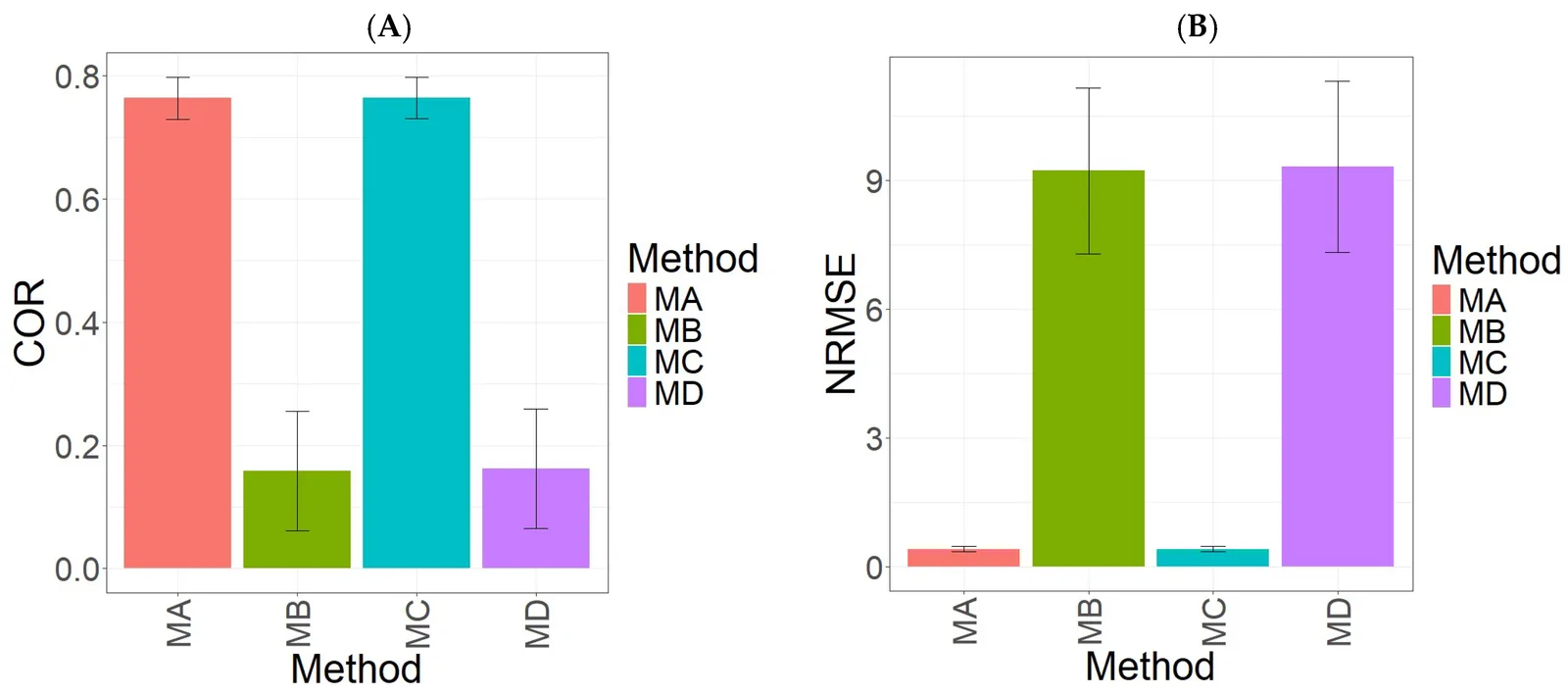
Comparative performance of prediction models for the Potato_Suecia_Rodomiro_Ortiz_2023 dataset. Panel (**A**) shows results based on Pearson’s correlation (COR), and Panel (**B**) shows results based on the normalized root mean squared error (NRMSE) for the multimodal predictor. Error bars represent the standard error across the 10 repeated 50% training–50% testing partitions (*n* = 10 per method within each dataset/trait summary); they quantify variation among repeated data splits, not posterior uncertainty. Models are defined in Section 4.2.

Results are shown in Figure 7 and Table A3. Models MC and MA achieved the highest correlation (COR = 0.764), whereas Model MB showed substantially lower performance (COR = 0.158). Descriptive ANOVA–Tukey summaries separated models with effective regularization across components from those including weakly regularized or unregularized components. The interpretation was supported by the large and consistent differences in COR and NRMSE. NRMSE values were low for Models MA and MC (NRMSE = 0.418), while Models MB and MD exhibited higher error. These results indicate that models with effective regularization across model components achieved more stable and accurate predictions than models in which regularization was absent or weakened in key components. Further details of these results are provided in Appendix D.

#### 2.2.3. Rice IRRI Philippines Spindel 2015 Multimodal

**Figure 8:**
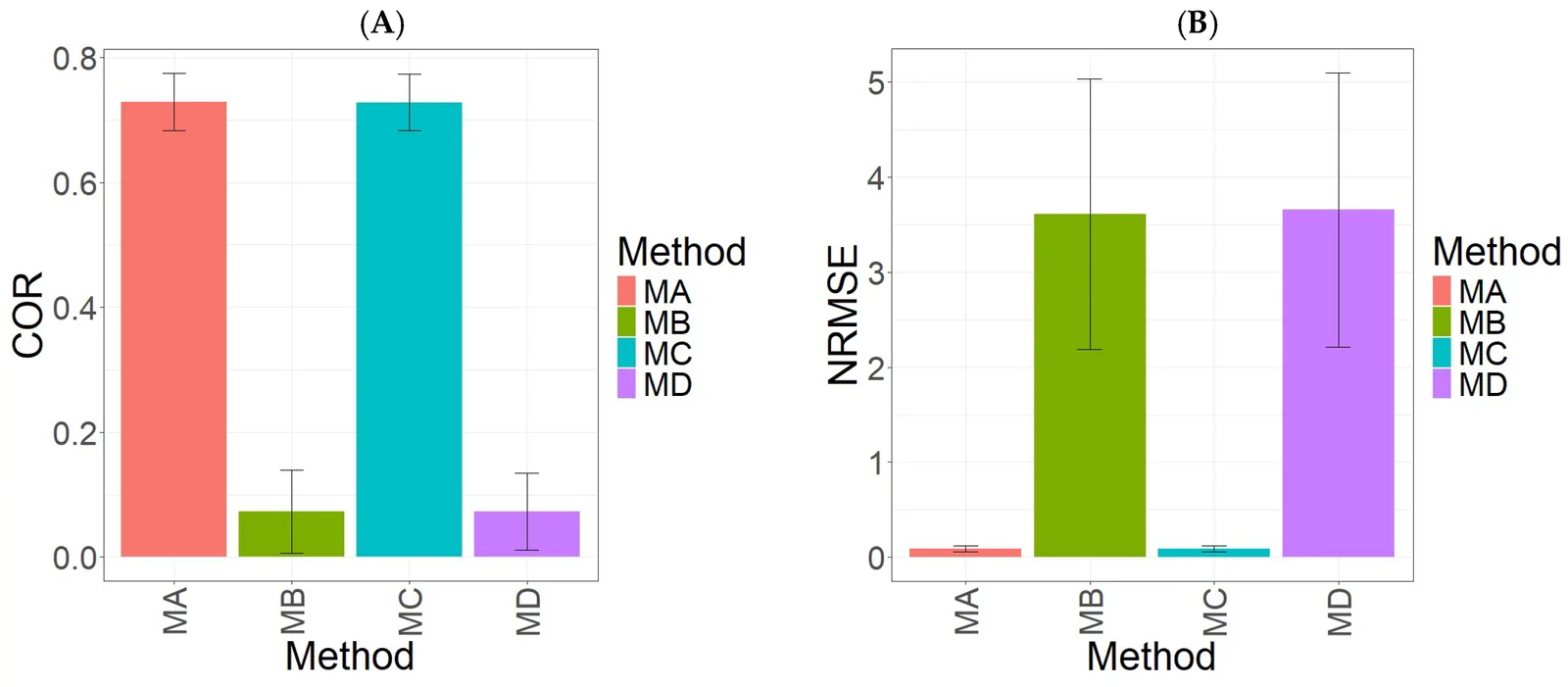
Comparative performance of prediction models for the Rice_IRRI_Philippines_Spindel_2015 dataset. Panel (**A**) shows results based on Pearson’s correlation (COR), and Panel (**B**) shows results based on the normalized root mean squared error (NRMSE) for the multimodal predictor. Error bars represent the standard error across the 10 repeated 50% training–50% testing partitions (*n* = 10 per method within each dataset/trait summary); they quantify variation among repeated data splits, not posterior uncertainty. Models are defined in Section 4.2.

Results are presented in Figure 8 and Table A3. Model MA achieved the highest correlation (COR = 0.729), followed closely by Model MC (COR = 0.728), while Models MB and MD showed substantially lower performance (COR ≈ 0.073). Descriptive ANOVA–Tukey summaries separated these configurations. Because repeated cross-validation observations are correlated, the interpretation was based primarily on the magnitude and consistency of the predictive differences. NRMSE values were low for Models MA and MC (NRMSE = 0.088), whereas Models MB and MD exhibited much higher error values. These results show that the largest performance differences were associated with the absence or weakening of regularization in multimodal model components, whereas models with effective regularization achieved substantially better predictive performance. Other details of these results are provided in Appendix D.

#### 2.2.4. Rice Kim 2020 Multimodal

**Figure 9:**
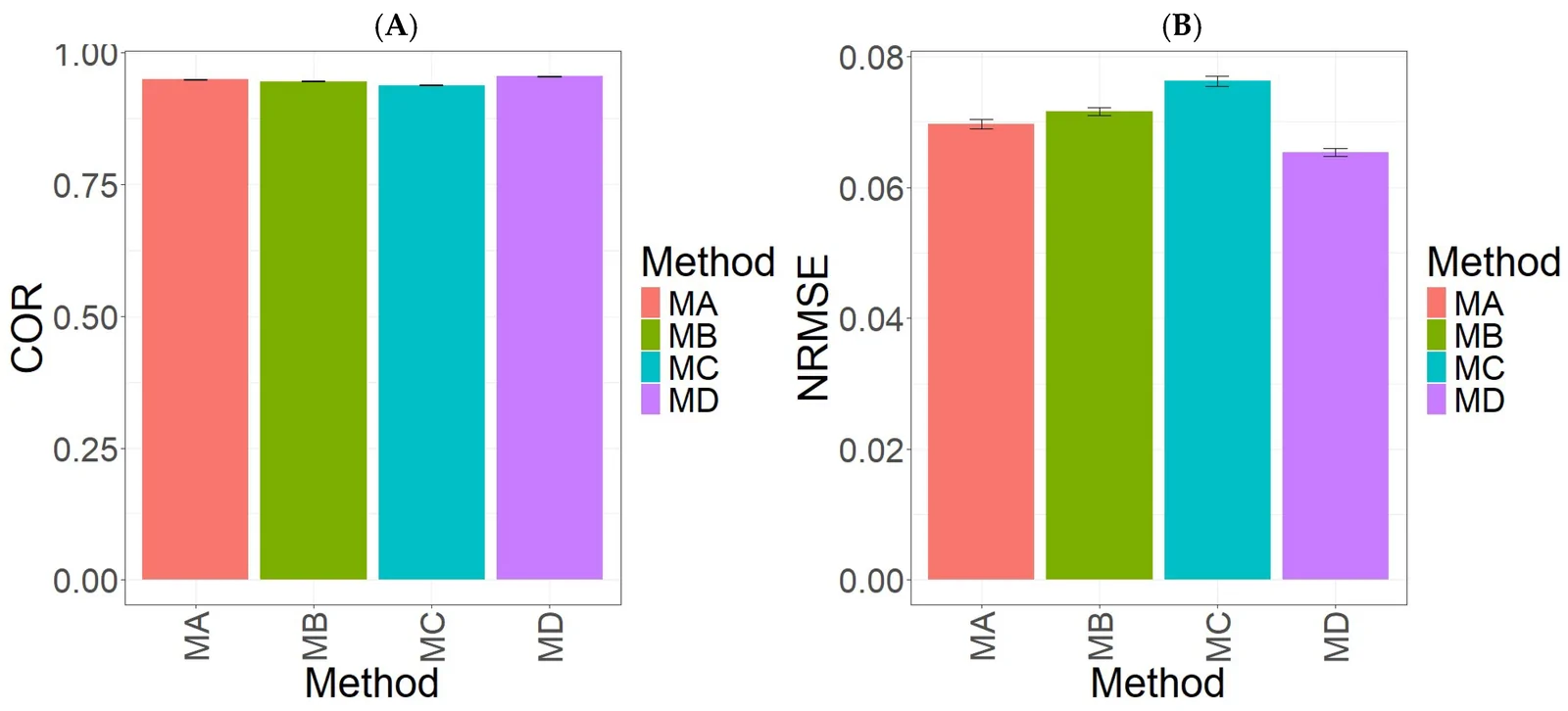
Comparative performance of prediction models for the Rice_Kim_2020 dataset. Panel (**A**) shows results based on Pearson’s correlation (COR), and Panel (**B**) shows results based on the normalized root mean squared error (NRMSE) for the multimodal predictor. Error bars represent the standard error across the 10 repeated 50% training–50% testing partitions (*n* = 10 per method within each dataset/trait summary); they quantify variation among repeated data splits, not posterior uncertainty. Models are defined in Section 4.2.

Results are shown in Figure 9 and Table A3. Model MD achieved the highest correlation (COR = 0.955), followed by Model MA (COR = 0.949). Differences among models in COR were small and not statistically significant. NRMSE values were similar across models (0.065–0.076), although statistically significant differences were detected for NRMSE. For this dataset, all multimodal models showed high predictive performance, with no significant differences in COR. This suggests that the impact of regularization structure was less pronounced in this dataset than in the other multimodal analyses. Full details of these results are provided in Appendix D.

#### 2.2.5. Summary Across Datasets Multimodal

**Figure 10:**
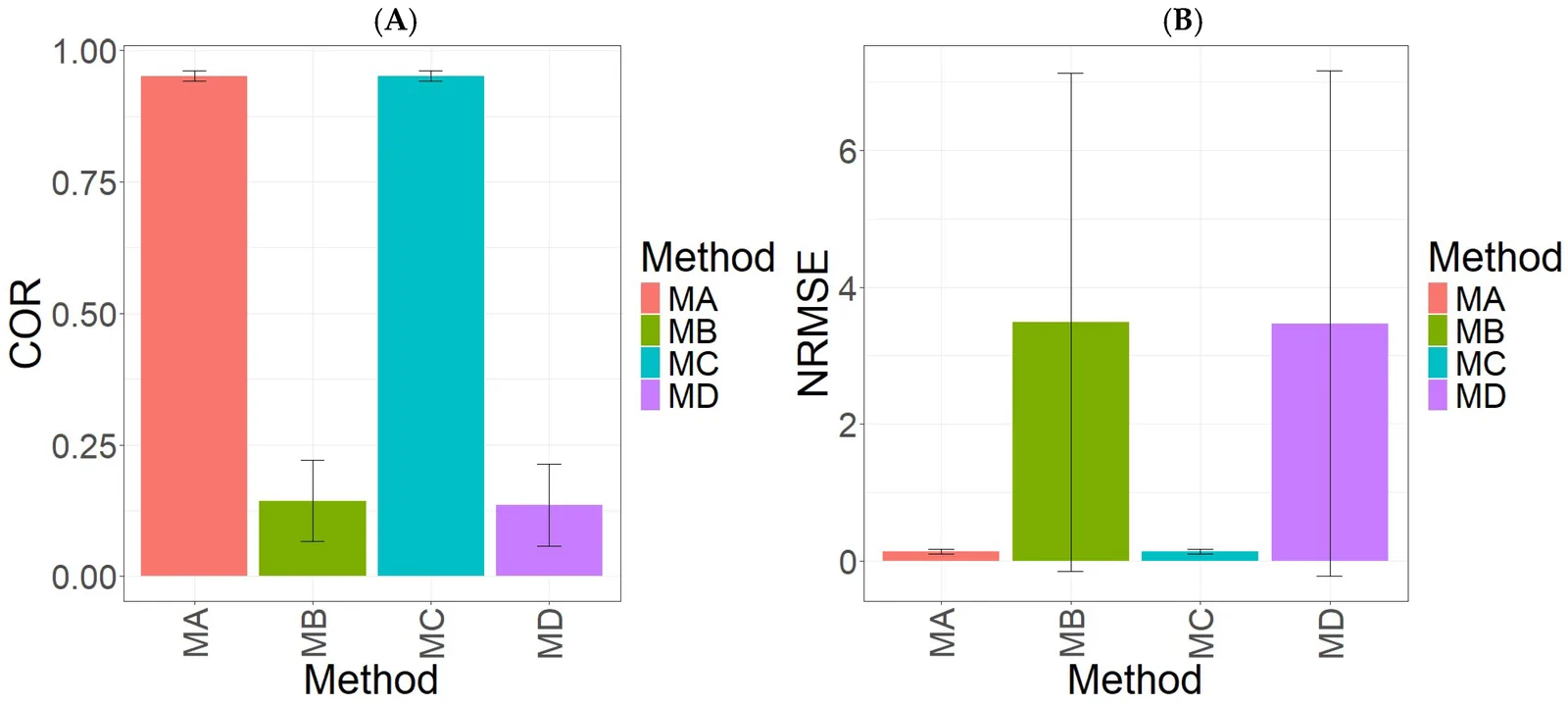
Comparative performance of prediction models for the summary across datasets. Panel (**A**) shows results based on Pearson’s correlation (COR), and Panel (**B**) shows results based on the normalized root mean squared error (NRMSE) for the multimodal predictor. Error bars represent the standard error across the 10 repeated 50% training–50% testing partitions (*n* = 10 per method within each dataset/trait summary); they quantify variation among repeated data splits, not posterior uncertainty. Models are defined in Section 4.2.

**Figure A3:**
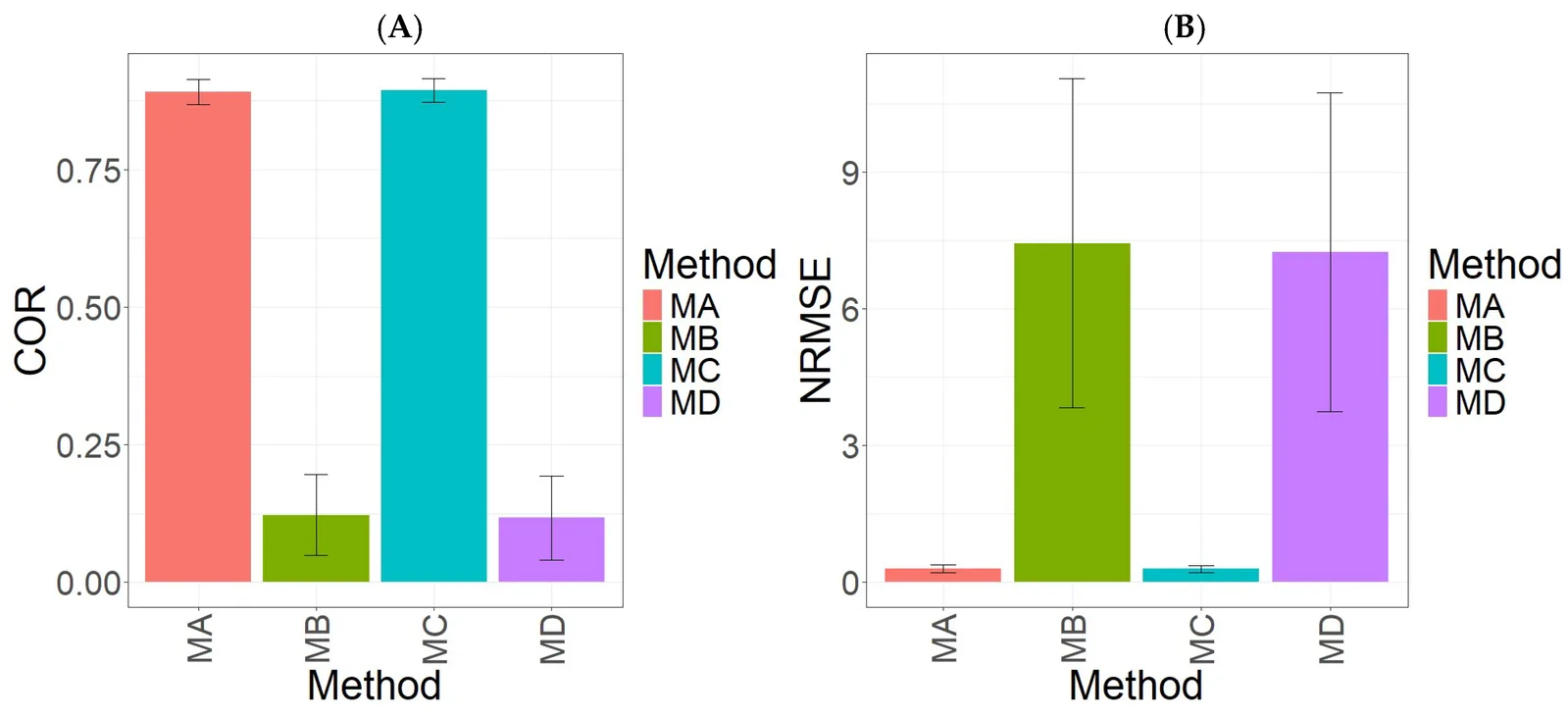
Comparative performance of prediction models for the Winter_Wheat_2020 dataset. Panel (**A**) shows results based on Pearson’s correlation (COR) and Panel (**B**) shows results based on the Normalized Root Mean Square Error (NRMSE). Model codes are defined in Section 4.2 and Table 3.

Combined multimodal results are shown in Figure 10 and Table A3. Models MA and MC achieved the highest predictive accuracy (COR ≈ 0.842), whereas Models MB and MD showed substantially lower performance (COR ≈ 0.143). Descriptive ANOVA–Tukey summaries separated these configurations. Because repeated cross-validation observations are correlated, the interpretation was based primarily on the magnitude and consistency of the predictive differences. NRMSE values were low for Models MA and MC (NRMSE ≈ 0.190), while Models MB and MD exhibited much higher error (NRMSE > 5). Across multimodal analyses, the main contrast was between models with coherent regularization across genomic, environmental, and G × E components and models in which regularization was absent or weakened in one or more components. Models MA and MC achieved the highest predictive accuracy and lowest prediction error, whereas Models MB and MD showed substantial degradation in performance. These results support the importance of prior-induced regularization for stable multimodal genomic prediction. However, because only a selected set of regularization configurations was evaluated, the results should not be interpreted as a complete comparison of all possible Bayesian prior structures. Additional details of these results are provided in Appendix D. For the remaining datasets not reported in this section, see Appendix E (Table A4 and Figure A3).

It is important to note that the summary results across datasets are based on simple unweighted averages of predictive performance metrics for both unimodal and multimodal models. Because the number of genotypes varies substantially among datasets, this aggregation approach may not fully reflect dataset-specific contributions. To address this limitation, results were also examined at the individual dataset level, and patterns were found to be consistent across crops, although some dataset-specific deviations were observed.

### 2.3. Variance Component Decomposition

To complement the understanding of the observed differences in predictive performance, we estimated variance components and broad-sense heritability (H) for each dataset under both unimodal and multimodal frameworks. The H was computed as H = σ^2^_g_/(σ^2^_g_ + σ^2^_e_/r) under unimodal models and as H = σ^2^_g_/(σ^2^_g_ + σ^2^_gE_/I + σ^2^_e_/Ir) under multimodal models, where I denotes the number of environments and r denotes the number of replications. It is also important to note that variance components and broad-sense heritability were estimated only for the UG model within the unimodal framework and for the MA model within the multimodal framework. These calculations were restricted to these models because the estimation of variance components requires assuming that all terms included in both unimodal and multimodal models are treated as random effects. These variance component estimates were used to complement the prediction results by examining how regularized model structures allocate variation across genomic, interaction, and residual components.

After auditing Appendix F, we confirmed that none of the reported variance-component estimates was negative. A small number were numerically close to zero and were interpreted as boundary estimates; they were treated as zero when summarizing broad-sense heritability. Environmental and G × E variance components were estimated only for the multimodal model, whereas the unimodal model included only the genomic predictor. Table 1 reports the resulting mean broad-sense heritability across the traits analyzed within each dataset.

Under the unimodal model (UG), the highest mean heritability values were observed in the two Winter_Wheat_Washington datasets, whereas the lowest was found in the Rice_Kim_2020 dataset. Notably, these datasets also corresponded to those with the highest and lowest prediction accuracies, respectively, under the UG framework. In contrast, within the multimodal model (MA), the lowest mean heritability was observed in the DMario_Family_14 dataset, while the highest was found in Rice_Kim_2020. Despite these differences, both datasets achieved high prediction accuracy, which may be attributed to the complexity of G × E interactions as well as the distinct biological nature of the evaluated traits. Detailed variance component estimates for each trait within each dataset are provided in Appendix F.

### 2.4. Results of Sensitivity Analysis

**Figure 11:**
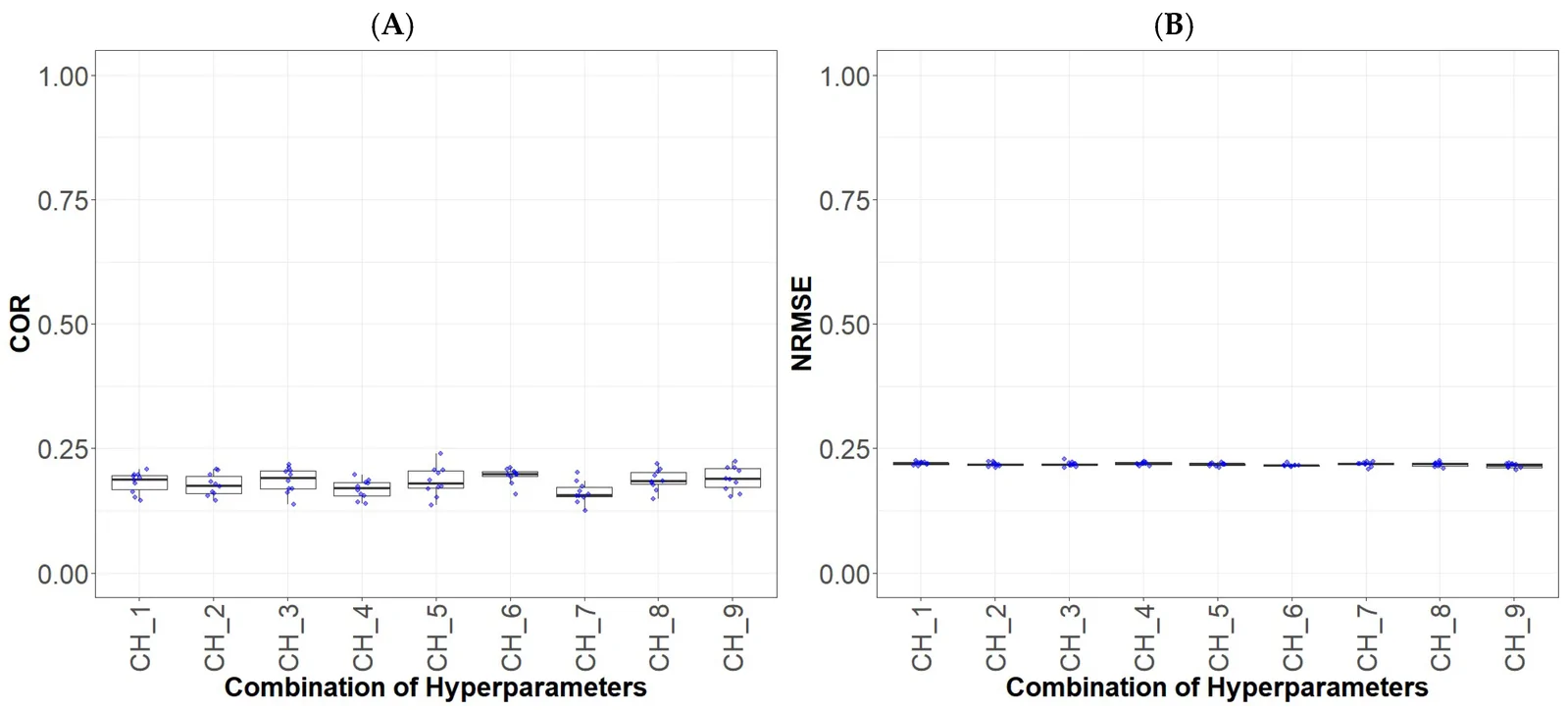
Dataset-specific sensitivity assessment for the nine hyperparameter combinations (CH) evaluated in the Rice_Kim_2020 dataset using the unimodal UG model. Panel (**A**) shows Pearson’s correlation (COR), and Panel (**B**) shows normalized root mean squared error (NRMSE).

Figure 11 presents a dataset-specific sensitivity assessment conducted on the Rice_Kim_2020 dataset using the unimodal UG model. The results indicate that, for this selected dataset and model, predictive performance was relatively stable across the nine hyperparameter combinations evaluated, as reflected by limited variation in both COR and NRMSE. These results should be interpreted as a limited sensitivity assessment rather than as evidence of general robustness across all datasets, crops, traits, and model classes. See details in Appendix G, Table A6.

**Figure 12:**
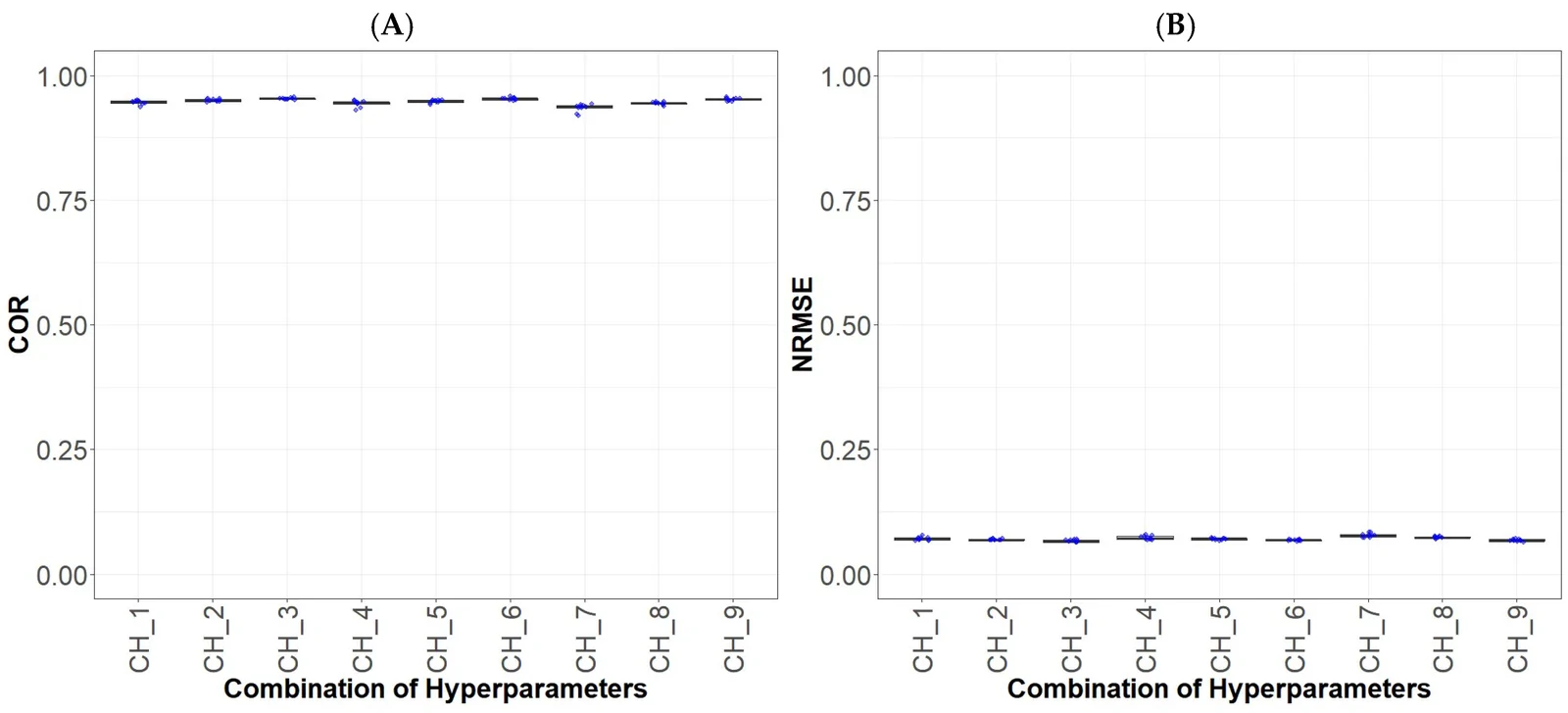
Dataset-specific sensitivity assessment for the nine hyperparameter combinations (CH) evaluated in the Rice_Kim_2020 dataset using the multimodal MA model. Panel (**A**) shows Pearson’s correlation (COR), and Panel (**B**) shows normalized root mean squared error (NRMSE).

Figure 12 presents a dataset-specific sensitivity assessment conducted on the Rice_Kim_2020 dataset using the multimodal MA model. The results indicate that, for this selected dataset and model, predictive performance was relatively stable across the nine hyperparameter combinations evaluated, with limited variation in both COR and NRMSE. As with the unimodal analysis, these findings should be interpreted as model- and dataset-specific rather than as a general robustness assessment for all multimodal models. See details in Appendix G, Table A7.

## 3. Discussion

Genomic selection (GS) has become a cornerstone of modern breeding programs, yet its success depends critically on the ability of statistical models to extract stable genetic signals from high-dimensional, noisy, and often unbalanced datasets. In this context, our results indicate that the dominant empirical contrast in this study was between regularized Bayesian genomic prediction models and weakly regularized or unregularized baseline formulations. Although the models differed in their prior structures, performance differences among regularized Bayesian models were generally modest. In contrast, the absence or weakening of regularization led to unstable estimation, inflated prediction error, and reduced predictive accuracy in several datasets. Therefore, the results should be interpreted primarily as evidence for the importance of prior-induced regularization in high-dimensional genomic prediction, rather than as a fine-scale ranking of alternative Bayesian prior families.

This distinction is important because the FIXED baseline used in this study should not be interpreted as a fully specified Bayesian non-informative prior in the strict sense. Rather, in the context of the present analyses, it functions as an unregularized or weakly regularized fixed-effect baseline. Consequently, the main comparison should be understood as regularization versus no or weak regularization. This clarification is essential because, in high-dimensional genomic prediction settings where the number of markers *p* greatly exceeds the number of observations *n*, the absence of regularization can lead to unstable parameter estimates, inflated residual variation, and poor predictive generalization.

From a statistical perspective, the observed behavior is consistent with the role of regularization in ill-posed estimation problems. In genomic prediction, *p* ≫ *n* is common, and many marker effects must be estimated from relatively limited phenotypic information. Under these conditions, weakly regularized or unregularized models may adapt excessively to noise in the training data, resulting in unstable predictions and large prediction errors. In contrast, regularized Bayesian models impose shrinkage on marker effects, constrain the parameter space, and improve the bias-variance trade-off [8,9]. Thus, the principal contribution of prior specification in these models is its ability to induce regularization, stabilize inference, and improve prediction reliability. Importantly, numerical stability, posterior convergence, and predictive stability are not interchangeable. Numerical instability was identified by extreme COR or NRMSE values and divergent predictions; posterior convergence was assessed from MCMC diagnostics; and predictive stability was evaluated from the dispersion and consistency of out-of-sample performance across repeated partitions.

The magnitude of the improvements observed in several datasets should therefore be interpreted carefully. In many cases, the large differences in predictive correlation and normalized root mean squared error were driven primarily by the poor performance of the weakly regularized or unregularized baseline, rather than by large differences among regularized Bayesian prior families. This interpretation is particularly important because models such as BRR, Bayes A, Bayes B, Bayes C, Bayesian Lasso, and RKHS-based formulations often showed relatively similar predictive performance. Therefore, the practical message is not that one specific Bayesian prior is universally superior, but that effective regularization is essential for stable genomic prediction in high-dimensional settings. Given the very large number of paired model–dataset–trait–partition comparisons, very small *p* values were expected even for modest differences. Accordingly, practical interpretation emphasized effect sizes and repeated consistency rather than statistical significance alone.

The comparison among regularized Bayesian models also provides useful information. BRR imposes homogeneous shrinkage across all marker effects and is often appropriate for highly polygenic traits, whereas Bayes A, Bayes B, Bayes C, and Bayesian Lasso allow different degrees of marker-specific shrinkage, variance heterogeneity, or variable selection [10,11,12]. RKHS regression provides an alternative form of regularization by placing a prior on functions through the kernel structure, allowing nonlinear relationships and complex genotype-phenotype patterns to be captured [13,16]. However, the results of this study suggest that, across many of the datasets evaluated, the presence of effective regularization was more influential than the specific choice among commonly used regularized Bayesian prior families. The importance of regularization was also evident in the multi-modal analyses. The multimodal genomic prediction models we implemented integrate genomic, environmental, and G × E components, thereby increasing model complexity and the number of variance components to be estimated [14,15]. In these settings, weak or absent regularization in one or more model components can lead to unstable variance allocation and degraded prediction. However, because the multimodal analysis considered only a selected set of regularization configurations, these results should not be interpreted as an exhaustive comparison of all possible prior structures. The variance component results further support the interpretation that regularized model structures can contribute to more stable variance allocation. In the unimodal and multimodal frameworks, broad-sense heritability was computed on an entry-mean basis as $H^{2}=\frac{\sigma_{g}^{2}}{\sigma_{g}^{2}+\frac{\sigma_{e}^{2}}{r}}$ for unimodal models and as $H^{2}=\frac{\sigma_{g}^{2}}{\sigma_{g}^{2}+\frac{\sigma_{gE}^{2}}{I}+\frac{\sigma_{e}^{2}}{Ir}}$ for multimodal models, where I is the number of environments and r is the number of replications.

The multimodal results can also be interpreted in terms of the location and extent of weak or absent regularization across model components. Model MB assigns weakly regularized or unregularized formulations to both genomic and environmental components, whereas model MD retains regularization for the environmental component and weakens it only for the genomic component. Consequently, instability in two major sources of variation provides a plausible explanation for the poorer performance of MB in several datasets. The four evaluated multimodal configurations were selected as representative regularization scenarios that isolate the contribution of genomic, environmental, and genotype-by-environment components. They should not be interpreted as an exhaustive benchmark of all possible Bayesian prior combinations; rather, they provide a controlled and mechanistically interpretable comparison of the consequences of removing regularization from specific model components. The inclusion of the two winter-wheat datasets is scientifically relevant rather than a source of unreliability. Wheat has a large, polyploid, and genetically complex genome, but genomic prediction is well established in wheat breeding and provides an informative test of model stability under complex genetic architecture. In the present study, predictive results were obtained for both Winter_Wheat_Washington datasets and are reported explicitly in Appendix C and Appendix E. Their inclusion broadens the empirical scope of the study and allows the regularization patterns observed in rice and potato to be assessed in a genetically more complex crop.

One important exception to the general pattern was observed for the Rice_Kim_2020 dataset. In this dataset, the weakly regularized or unregularized baseline performed competitively, and in some cases achieved slightly higher predictive correlation than regularized Bayesian alternatives. This result emphasizes that the benefits of regularization may depend on dataset structure, trait architecture, marker redundancy, and signal-to-noise ratio. One possible explanation is that strong marker correlation or redundancy may reduce the effective dimensionality of the predictor space, allowing simpler models to perform competitively. However, because we did not directly estimate linkage disequilibrium patterns, matrix condition numbers, eigenvalue decay, or effective rank, this interpretation should be considered a hypothesis rather than a demonstrated mechanism. Additional empirical diagnostics would be required to support this explanation. Therefore, the Rice_Kim_2020 dataset should be regarded as an informative exception showing that the benefit of regularization may depend on dataset structure, marker redundancy, trait architecture, and signal-to-noise ratio.

From a practical breeding perspective, the main implication is that high-dimensional genomic prediction models should include effective regularization. Breeding datasets frequently involve many more markers than individuals, unbalanced multi-environment trials, and traits controlled by many small-effect loci. In such contexts, unregularized or weakly regularized models may produce unstable genomic estimated breeding values and inflated prediction error. Regularized Bayesian genomic prediction models can reduce this risk by stabilizing marker-effect estimates and improving prediction reliability. Nevertheless, the results do not imply that one Bayesian prior family is universally optimal. Prior choice should be guided by trait architecture, dataset size, marker structure, computational feasibility, and the objectives of the breeding program.

The sensitivity analyses should also be interpreted cautiously. They provided a dataset-specific assessment of selected hyperparameter combinations for the Rice_Kim_2020 dataset and selected models. The relatively stable performance observed in those analyses suggests that, for that specific dataset and model configuration, prediction was not highly sensitive to the evaluated hyperparameter combinations. However, these analyses should not be interpreted as evidence of general robustness across all datasets, crops, traits, and model classes. Broader sensitivity analyses would be required to draw general conclusions about hyperparameter robustness in unimodal and multimodal Bayesian genomic prediction. Second, the FIXED baseline should be interpreted as an unregularized or weakly regularized model rather than as a formal Bayesian non-informative prior. Third, the cross-dataset summaries were based on simple averages and may not fully reflect differences in genotype number, marker number, trait number, or crop-specific structure across datasets. Fourth, the sensitivity analysis was restricted to models UG and MA in the Rice_Kim_2020 dataset and should therefore be interpreted as a model- and dataset-specific assessment rather than as evidence of robustness across all datasets and model classes. Finally, mechanistic explanations for dataset-specific exceptions, such as Rice_Kim_2020, require additional empirical diagnostics such as linkage disequilibrium summaries, matrix condition numbers, eigenvalue decay, or effective rank.

Overall, our findings indicate that prior-induced regularization was associated with improved predictive behavior in most of the evaluated high-dimensional datasets, particularly relative to the weakly regularized or unregularized baseline. The evidence is strongest for the selected genomic and environmental configurations that were directly compared; because RKHS was retained for G × E in every multimodal model, the study does not independently establish the optimal regularization strategy for that component. Conclusions therefore emphasize effect magnitude, consistency across repeated validation partitions, and dataset-specific exceptions rather than significance tests alone.

Results show that prior-induced regularization is an important component of model design in Bayesian genomic prediction. The largest empirical differences were observed between regularized models and weakly regularized or unregularized baselines, rather than among regularized Bayesian prior families themselves. Therefore, priors should be selected and justified not only as Bayesian assumptions, but also as regularization mechanisms that influence shrinkage, variance allocation, model stability, and predictive reliability. For breeding applications, these results support the use of appropriately regularized Bayesian models to improve prediction stability, reduce error inflation, and enhance the reliability of genomic prediction in high-dimensional unimodal and multimodal settings.

## 4. Materials and Methods

### 4.1. Datasets

In Table 2, we provide information on the six datasets used in this study.

The datasets used in this study originate from previously published experiments conducted under different conditions and experimental designs, including multi-environment trials and structured breeding populations. While most datasets were generated under controlled experimental designs such as randomized or partially balanced trials, the level of balance, environmental heterogeneity, and data completeness varies across studies. The datasets also differ biologically and technically: wheat is an allohexaploid cereal, rice is diploid, and cultivated potato is an autotetraploid crop; their population structures, trait architectures, marker densities, and experimental designs are therefore not directly equivalent. Marker matrices were analyzed using the coding supplied with each published dataset and standardized within each dataset before kernel construction. Consequently, comparisons are interpreted primarily within dataset and across common predictive metrics, rather than as evidence that a single biological architecture or preprocessing assumption is identical across species.

Because this study relies on publicly available datasets, a formal quality scoring was not applied. However, differences in experimental design, data structure, and noise levels are expected to influence predictive performance. Therefore, results should be interpreted in the context of dataset-specific characteristics rather than assuming complete comparability across all cases.

Genotypic data were preprocessed using standard quality control procedures. SNP markers with minor allele frequency (MAF) lower than 0.05 and markers with more than 10% missing values were removed. Missing genotypes were imputed using mean allele frequencies. Marker matrices were subsequently centered and standardized prior to model fitting to ensure comparability across datasets.

#### Details of Phenotypic Datasets Used in This Study

Phenotypic data were obtained from previously published and curated datasets corresponding to the original studies cited in Table 1. When adjusted phenotypic values such as BLUEs or BLUPs were available from the original publications, these values were used directly for genomic prediction analyses. No missing data issues were observed in the available phenotypic datasets; therefore, no additional outlier removal or phenotype filtering was applied beyond the preprocessing procedures described in the original studies.

Because the datasets originated from independent studies with different experimental designs, phenotypic preprocessing protocols were not identical across datasets; however, all datasets were analyzed using standardized genomic prediction pipelines after preprocessing.

### 4.2. Statistical Models

Seven unimodal models were considered and are denoted as UA, UB, UC, UD, UE, UF, and UG. For multimodal models only four models were considered, denoted as MA, MB, MC and MD, resulting in a total of 11 models.

Throughout the model notation, the superscript ^T^ denotes matrix or vector transpose and is not an exponent or power parameter.

This model was considered to be:

(1) $$Y_{ij}=\mu +\sum_{k=1}^{p}x_{jk}\beta_{k}+\varepsilon_{ij}$$

Method UA corresponds to the FIXED formulation and was used as a weakly regularized or unregularized fixed-effect baseline for marker-effect coefficients. It represents the absence of effective prior-induced shrinkage and was included to quantify the empirical effect of regularization relative to the Bayesian models.

Method UB uses Bayesian Ridge Regression (BRR), method UC uses Bayes A, method UD uses Bayes B, method UE uses Bayes C, method UF uses the Bayesian Lasso (BL), and method UG uses Reproducing Kernel Hilbert Space (RKHS) regression. These seven unimodal models were implemented in the BGLR package in R [12]. Their prior distributions and computational specifications are summarized in Table 3.

In summary, U denotes unimodal models and M denotes multimodal or multi-component models. The second letters A–G identify the prior/model specification. For unimodal models, UA–UG denote FIXED, BRR, Bayes A, Bayes B, Bayes C, Bayesian Lasso, and RKHS, respectively. For multimodal models, MA–MD denote selected combinations of FIXED and RKHS specifications for the environmental, genomic, and G × E components.

In this study, unimodal models use genomic information only, whereas multimodal (multi-component) models integrate genomic, environmental, and genotype-by-environment components. This terminology refers to the number of information components included in the predictor, not to the number of environments. The interpretive rationale concerning differences in regularization among these model configurations is presented in the Discussion.

The multimodal analysis considered four selected regularization configurations, defined below and summarized in Table 4.

#### 4.2.1. Multimodal (M) Predictors

The multimodal analysis considered a selected set of regularization configurations rather than all possible prior combinations. In all four configurations, the G × E component was modeled using RKHS; only the regularization assigned to the genomic and environmental components varied. Therefore, this design permits comparison of those selected configurations but not estimation of an independent main effect of G × E regularization or a complete factorial decomposition of regularization choices across components.

This model is equal to:

(2) $$Y_{ij}=\mu +E_{i}+\sum_{k=1}^{p}x_{jk}\beta_{k}+\sum_{k=1}^{p}x_{ijk}\beta_{ik}+\varepsilon_{ij}$$

All terms are the same as those defined in model (1), with the exception of the terms $E_{i}$ i=1,…,I, that denotes the random effects of environments distributed as $E={\left(E_{1},\ldots ,E_{I}\right)}^{T}∼N_{I}\left(0,\sigma_{E}^{2}I\right)$, where I, is an identity matrix of order I and the term $\sum_{k=1}^{p}x_{ijk}\beta_{ik}$ that corresponds to the marker by environment interaction; for this reason $\beta_{ik}$ denotes the marker effect k at environment i. Also, each $\beta_{ik}$ follows any of the distributions given in Table 2. When model (2) is reparametrized as a GBLUP model the term $g_{j}=\sum_{k=1}^{p}x_{jk}\beta_{k},$ described in model 1, denotes the random effects of genotypes and the term ${gE}_{ij}=\sum_{j=1}^{p}x_{ijk}\beta_{ik}$ denotes the G × E interaction effects, $gE={\left(gE_{11},\ldots ,gE_{1J},\ldots ,\;gE_{IJ}\right)}^{T}$ that are modeled as following a multivariate normal distribution $gE∼N_{IJ}\left(0,\sigma_{gE}^{2}\left(K_{g}{}^{\circ}K_{E}\right)\right)$, where ${K_{g}=Z}_{g}GZ_{g}^{T}$, $Z_{g}$ is the incidence matrix for the additive genetic effects and G denotes the genomic relationship matrix [19]; the variance component $\sigma_{gE}^{2}$ corresponds to the G × E interaction, $K_{E}=\frac{Z_{E}Z_{E}^{T}}{I}\;$ represents the linear kernel of the environments, and $Z_{E}$ is the incidence matrix representing the environmental effects. ° denotes the Hadamard product. The Hadamard product $K_{g}{}^{\circ}K_{E}$ reflects the variance-covariance of the **G × E** interaction effects, because: (a) ${\left(K_{g}{}^{\circ}K_{E}\right)}_{ij}$ = ${K_{g}}_{ij}{K_{E}}_{ij}$ is high only when both ${K_{g}}_{ij}$ (genetic similarity) and ${K_{E}}_{ij}$ (environmental similarity) are high, where ${K_{g}}_{ij}$ denotes (i,j) entry of a matrix $K_{g}$ and (b) It captures joint similarity, so it models effects that are specific to combinations of genetic and environmental contexts. It is important to point out that since both $K_{g}$ and $K_{E}$ are symmetric and positive semi-definite (PSD) variance-covariance matrices, the Hadamard product ($K_{g}{}^{\circ}K_{E})$ is a valid variance-covariance matrix. It is important to point out that for the implementation of model (2) was also performed in the BGLR R statistical software [12] and here we only generated four methods by mixing RKHS (informative prior) and the FIXED non-informative prior. The four predictors resulting of implementing model (2) are provided in Table 3.

For the multimodal models, RKHS was used as the regularized specification and FIXED as the weakly regularized or unregularized specification. The four combinations in Table 4 were selected to compare regularization across genomic, environmental, and genotype-by-environment components.

**Table 4:** Composition of the predictions of model (2) implemented in BGLR.

Method	Environment	Genotypes (G)	G × E
MA	RKHS	RKHS	RKHS
MB	FIXED	FIXED	RKHS
MC	FIXED	RKHS	RKHS
MD	RKHS	FIXED	RKHS

Table 4 summarizes how regularized (RKHS) and weakly regularized or unregularized fixed-effect baseline (FIXED) priors are combined across genomic, environmental, and G × E components to define the multimodal models (MA–MD). To be more specific it is important to point out that in model MA, the environments (E) are assumed to be distributed as $N_{I}\left(0,\sigma_{E}^{2}I_{I}\right)$, the genotypes are distributed as $N_{J}\left(0,\sigma_{g}^{2}G\right)$, and the G × E interaction effects are distributed as $N_{IJ}\left(0,\sigma_{gE}^{2}\left(K_{g}{}^{\circ}K_{E}\right)\right)$. While for model MB in Equation (2), each environmental effect is assumed to be distributed as $N\left(0,10,000\right)$, that is, they had a weakly regularized or unregularized prior, while for genotype effects each beta coefficient of markers, $\beta_{k}$, also is assumed to be $N\left(0,10,000\right),$ and the G × E random interaction effects are distributed as $N_{IJ}\left(0,\sigma_{gE}^{2}\left(K_{g}{}^{\circ}K_{E}\right)\right)$. For model MC environment effects are assumed to be distributed as $N\left(0,10,000\right)$, genotypes are assumed distributed as $N_{J}\left(0,\sigma_{g}^{2}G\right)$ and G × E effects are assumed to be distributed as $N_{IJ}\left(0,\sigma_{gE}^{2}\left(K_{g}{}^{\circ}K_{E}\right)\right)$. Finally for model MD environments are assumed to be distributed as $N_{I}\left(0,\sigma_{E}^{2}I_{I}\right)$, for genotypes the beta coefficients of each marker effects, $\beta_{k}$, are assumed to be $N\left(0,10,000\right),$ and the G × E effects are assumed to be distributed as $N_{IJ}\left(0,\sigma_{gE}^{2}\left(K_{g}{}^{\circ}K_{E}\right)\right)$.

Although additional combinations of regularized and weakly regularized or unregularized baseline formulations across genomic, environmental, and G × E components could be explored, the objective of this study was not to perform an exhaustive combinatorial comparison of all possible prior configurations. Instead, the multimodal framework was intentionally designed as a controlled regularization experiment aimed at evaluating the consequences of removing regularization from specific model components while maintaining mechanistic interpretability.

#### 4.2.2. Implementation Details and Hyperparameter Specification

All Bayesian models were implemented using the BGLR package in R. For all analyses, we used a Markov Chain Monte Carlo (MCMC) algorithm with 30,000 iterations, including a burn-in period of 10,000 iterations and a thinning interval of 5 to reduce autocorrelation.

Hyperparameters were specified following standard recommendations in the BGLR framework. For variance components, scaled inverse chi-squared priors were defined with degrees of freedom ν = 5 and scale parameters derived from the phenotypic variance of each dataset. For the Bayesian Lasso, the shrinkage parameter λ was assigned a Gamma(1, 0.0001) prior. For Bayes B and Bayes C models, the prior proportion of non-zero effects (π) was specified using a Beta(1, 1) distribution. These values correspond to weakly informative but proper priors and ensure comparable levels of regularization across models.

#### 4.2.3. MCMC Convergence and Implementation

All Bayesian models were implemented using the BGLR package with 30,000 MCMC iterations, a burn-in period of 10,000 iterations, and thinning every 5 samples. Convergence was evaluated by inspecting the post-burn-in trace behavior and the stability of posterior summaries. These checks were used to identify evident nonstationarity, poor mixing, or numerical divergence. Because formal multi-chain diagnostics were not available uniformly for every model–dataset–trait combination, no universal Gelman–Rubin or effective-sample-size threshold is claimed.

Posterior convergence should therefore be distinguished from predictive and numerical stability: the former concerns the behavior of the sampled posterior after burn-in, predictive stability concerns the consistency of out-of-sample COR and NRMSE across repeated partitions, and numerical stability concerns the absence of extreme or divergent predictions.

Hyperparameters for prior distributions were specified following standard recommendations in the BGLR framework. For variance components, scaled inverse chi-squared priors were used with degrees of freedom and scale parameters chosen to provide weakly informative but proper regularization. For the Bayesian Lasso, the shrinkage parameter λ was assigned a gamma prior, while for Bayes B and Bayes C models, the prior proportion of non-zero effects (π) was specified using a beta distribution. In all cases, default settings were used unless otherwise indicated, ensuring comparability across models. Very specific details of the priors for each of the Bayesian models implemented can be found in the supplemental materials of the paper of Pérez and de Los Campos [12].

All analyses were conducted using R statistical software (version 4.6.0) and model fitting was performed under identical cross-validation partitions to ensure a fair comparison of predictive performance across methods.

### 4.3. Sensitivity Analysis

We conducted a sensitivity analysis (Table 5) using the Rice_Kim_2020 dataset, focusing exclusively on the UG and MA models. To assess the impact of the hyperparameter settings, we evaluated the predictive performance of both models under the nine different combinations of two hyperparameters considered in this study.

It is important to note that R2 represents the prior belief regarding the proportion of phenotypic variance explained by the model. Larger values of R2 imply a stronger prior assumption that the predictors are highly informative and account for a substantial fraction of the phenotypic variation. Consequently, values such as R2 = 0.1 are generally considered weakly informative, R2 = 0.5 moderately informative, and R2 = 0.9 highly informative. The second hyperparameter, df, denotes the degrees of freedom of the scaled inverse chi-square prior assigned to the genetic variance component. This parameter controls the strength of the prior distribution, with smaller values yielding more diffuse priors and larger values producing more concentrated priors. In practice, df = 3 is often regarded as weakly informative, df = 5 as moderately informative, and df = 20 as strongly informative. The sensitivity analysis was restricted to models UG and MA in the Rice_Kim_2020 dataset to illustrate their response to substantial changes in the selected hyperparameters. Accordingly, it should be interpreted as a model- and dataset-specific assessment rather than as evidence of robustness across all models, datasets, crops, and traits.

### 4.4. Cross-Validation Strategies and Metrics

For both predictors, we evaluated the prediction performance of the different priors listed in Table 2 using a cross-validation scheme of untested lines in tested environments. Specifically, we randomly removed 50% of the lines to form the testing set, while the remaining 50% were used as the training set to fit the models [20]. This data partitioning process was repeated 10 times, and the average prediction accuracy was computed for each dataset and evaluation metric.

Predictive performance was assessed using NRMSE and Pearson’s correlation (COR), calculated between observed and predicted values in each testing partition. Means and standard errors were summarized across the 10 repeated 50% training–50% testing partitions. The same partitions were used for every method, preserving paired comparisons within dataset, trait, and repetition. ANOVA followed by Tukey grouping was retained only as a complementary descriptive summary of each model–dataset–trait–partition comparison. Repeated cross-validation observations are correlated, and the very large number of comparisons can make small effects appear highly significant. Accordingly, omnibus significance was reported only at the threshold level when exact values were not available from the archived summaries, and no conclusion was based on *p* values or Tukey letters alone. Interpretation emphasized effect magnitude, consistency across paired partitions, agreement between COR and NRMSE, and the presence or absence of extreme predictions.

## 5. Conclusions

This study provides empirical evidence that prior-induced regularization is an important determinant of predictive accuracy and stability when regularized Bayesian models are compared with weakly regularized or unregularized high-dimensional baselines. Across six datasets from wheat, rice, and potato, the largest differences were generally observed between models with effective shrinkage and models lacking sufficient regularization, rather than among the regularized Bayesian prior families themselves. The results therefore support the conclusion that effective regularization is essential for stable genomic prediction when the number of markers greatly exceeds the number of phenotyped individuals.

For plant-breeding applications, appropriately regularized models can reduce error inflation and improve the reliability of genomic estimated breeding values. This conclusion is supported by the unimodal and multimodal results, including the two winter-wheat datasets. In multimodal prediction, coherent regularization across genomic, environmental, and genotype-by-environment components was particularly important for stable variance allocation and reliable prediction.

Nevertheless, the findings do not establish that one Bayesian prior family is universally optimal. Prior choice should be aligned with trait architecture, dataset structure, marker dimensionality, model complexity, and breeding objectives. The multimodal analysis evaluated representative rather than exhaustive prior configurations, and Rice_Kim_2020 demonstrated that weakly regularized or unregularized formulations may occasionally remain competitive. Future studies should examine additional prior combinations, crop-specific summaries, and empirical diagnostics of marker redundancy and effective dimensionality.

## Figures and Tables

**Table 1:** Mean broad-sense heritability (Mean_H) and its standard deviation (sd_H) across traits for each dataset under models MA and UG. n_traits denotes the number of traits.

Dataset	Model	Mean_H	sd_H	n_traits
DMario_Family_14	MA	0.377	0.541	3
DMario_Family_14	UG	0.602	0.381	3
Potato_Suecia_Rodomiro_Ortiz_2023	MA	0.405	0.090	7
Potato_Suecia_Rodomiro_Ortiz_2023	UG	0.473	0.134	7
Rice_IRRI_Philippines_Spindel_2015	MA	0.532	0.237	18
Rice_IRRI_Philippines_Spindel_2015	UG	0.495	0.220	18
Rice_Kim_2020	MA	0.810	-	1
Rice_Kim_2020	UG	0.204	-	1
Winter_Wheat_Washington_2020	MA	0.422	0.410	16
Winter_Wheat_Washington_2020	UG	0.890	0.103	16
Winter_Wheat_Washington_2021	MA	0.115	0.059	16
Winter_Wheat_Washington_2021	UG	0.878	0.122	16

**Table 2:** For each dataset, the number of environments (Env), genotypes (lines), molecular markers, and traits included in the analytical summaries is reported, together with the corresponding reference. The audited trait counts are consistent with Table 1 and Appendix F.

Number	Dataset Name	No. Env	No. Lines	No. Markers	No. Traits	Reference
1	DMario_Family_14	12	532	2744	3	[16]
2	Potato_Suecia_Rodomiro_Ortiz_2023	6	253	11,080	7	[14]
3	Rice_IRRI_Philippines_Spindel_2015	2	319	13,101	18	[15]
4	Rice_Kim_2020	8	107	108,024	1	[17]
5	Winter_Wheat_Washington_2020	6	758	16,382	16	[18]
6	Winter_Wheat_Washington_2021	8	456	16,382	16	[18]

**Table 3:** Prior densities implemented in BGLR.

Model	Join Distribution of Effects and Hyper-Parameters
FIXED	$p(\beta_{k})\propto 1$ equivalent to $N\left(0,10,000\right)$
BRR	$p\left(\beta_{k},\;\sigma_{\beta }^{2}\right)=\left\{\prod_{l}N\left(\beta_{kl}|0,\sigma_{\beta }^{2}\right)\right\}\chi^{-2}(\sigma_{\beta }^{2}|df_{\beta },S_{\beta })$
BayesA	$p\left(\beta_{k},\sigma_{\beta_{k}}^{2},S_{\beta }\right)=\left\{\prod_{l}N(\beta_{kl}|0,\sigma_{\beta_{kl}}^{2})\chi^{-2}(\sigma_{\beta_{kl}}^{2}|df_{\beta },S_{\beta })\right\}G(S_{\beta }|r,s)$
BL	$p(\beta_{k},\tau_{k}^{2},\lambda^{2}|\sigma_{\varepsilon }^{2})=\left\{\prod_{l}N\left(\beta_{kl}|0,\tau_{kl}^{2}\times \sigma_{\varepsilon }^{2}\right)Exp\left\{\tau_{kl}^{2}|\frac{\lambda^{2}}{2}\right\}\right\}\times G\left(\lambda^{2}|r,s\right),\;or$ $p(\beta_{k},\tau_{k}^{2},\lambda |\sigma_{\varepsilon }^{2},max)=\left\{\prod_{l}N\left(\beta_{kl}|0,\tau_{kl}^{2}\times \sigma_{\varepsilon }^{2}\right)Exp\left\{\tau_{kl}^{2}|\frac{\lambda^{2}}{2}\right\}\right\}\times B\left(\lambda /max|p_{0,}\pi_{0}\right),\;or$ $p(\beta_{k},\tau_{k}^{2}|\sigma_{\varepsilon }^{2},\lambda )=\left\{\prod_{l}N\left(\beta_{kl}|0,\tau_{kl}^{2}\times \sigma_{\varepsilon }^{2}\right)Exp\left\{\tau_{kl}^{2}|\frac{\lambda^{2}}{2}\right\}\right\}$
BayesC	$p(\beta_{k},\sigma_{\beta }^{2},\pi )=\left\{\prod_{l}\left[\pi N\left(\beta_{kl}|0,\sigma_{\beta }^{2}\right)+\left(1-\pi \right)1\left(\beta_{kl}=0\right)\right]\right\}\times \chi^{-2}\left(\sigma_{\beta }^{2}|df_{\beta },S_{\beta }\right)B\left(\pi |p_{0},\pi_{0}\right)$
BayesB	$p(\beta_{j},\sigma_{\beta }^{2},\pi )=\left\{\prod_{l}\left[\pi N\left(\beta_{kl}|0,\sigma_{\beta }^{2}\right)+\left(1-\pi \right)1\left(\beta_{kl}=0\right)\right]\chi^{-2}\left(\sigma_{\beta_{kl}}^{2}|df_{\beta },S_{\beta }\right)\right\}B\left(\pi |p_{0},\pi_{0}\right)\times G\left(S_{\beta }|r,s\right)$
RKHS	$p\left(u_{l},\sigma_{u_{l}}^{2}\right)=N\left(u_{l}|0,K_{l}\times \sigma_{u_{l}}^{2}\right)\chi^{-2}\left(\sigma_{u_{l}}^{2}|df_{l},S_{l}\right)$

$N\left(\cdot |\cdot ,\cdot \right),{\;\chi }^{-2}\left(\cdot |\cdot ,\cdot \right),\;G\left(\cdot |\cdot ,\cdot \right),\;Exp\left(\cdot |\cdot \right),\;B\left(\cdot |\cdot ,\cdot \right)$ denote normal, scaled inverse Chi-squared, gamma, exponential and beta densities, respectively. max is a user-defined parameter representing the maximum value that λ can take and $\pi_{0}$ is the prior proportion of non-zero effects [12].

**Table 5:** Sensitivity analysis using the Rice_Kim_2020 dataset for models UG and MA for two hyperparameters: degrees of freedom (df) of the scaled inverse chi-square prior assigned to the genetic variance component and the proportion of phenotypic variance explained by the model (R2). CH denotes hyperparameter combination.

CH	df	R2
CH_1	3	0.1
CH_2	3	0.5
CH_3	3	0.9
CH_4	5	0.1
CH_5	5	0.5
CH_6	5	0.9
CH_7	20	0.1
CH_8	20	0.5
CH_9	20	0.9

## Data Availability

Data are available in a publicly accessible repository. The original data presented in the study are openly available at the following GitHub repository: https://github.com/eliaspechav/Uni-Multi-Inputs-on-GS (accessed on 31 July 2026). This repository also contains all the R scripts used for the implementation of the unimodal and multimodal models.

## Abbreviations

BGP	Bayesian genomic prediction
BL	Bayesian Lasso
BRR	Bayesian ridge regression
COR	Pearson correlation
FIXED	weakly regularized or unregularized fixed-effect baseline
G	genomic component
G × E	genotype-by-environment interaction
GS	genomic selection
MA–MD	multimodal model configurations
MCMC	Markov chain Monte Carlo
NRMSE	normalized root mean squared error
RKHS	reproducing kernel Hilbert space
SNP	single nucleotide polymorphism
UA–UG	unimodal model configurations.

## Appendix B

**Table A1:** Prediction performance for the unimodal predictor (U) of evaluated models in terms of Pearson’s correlation (COR) and Normalized Root Mean Square Error (NRMSE) for the DMario_Family, Potato_Suecia, Rice_IRRI, Rice_Kim and across datasets. Tukey grouping letters summarize the descriptive ANOVA comparisons. Because repeated cross-validation observations are correlated, the letters should be interpreted together with effect magnitude and consistency across partitions, not as stand-alone confirmatory inference.

	Dataset	Methods	COR	NRMSE
1	DMario_Family_14	UA	0.337 ^a^	2.539 ^b^
2	DMario_Family_14	UB	0.476 ^a^	0.106 ^a^
3	DMario_Family_14	UC	0.461 ^a^	0.106 ^a^
4	DMario_Family_14	UD	0.462 ^a^	0.106 ^a^
5	DMario_Family_14	UE	0.471 ^a^	0.106 ^a^
6	DMario_Family_14	UF	0.462 ^a^	0.106 ^a^
7	DMario_Family_14	UG	0.466 ^a^	0.106 ^a^
1	Potato_Suecia_Rodomiro_Ortiz_2023	UA	0.097 ^b^	16.489 ^b^
2	Potato_Suecia_Rodomiro_Ortiz_2023	UB	0.350 ^a^	0.626 ^a^
3	Potato_Suecia_Rodomiro_Ortiz_2023	UC	0.343 ^a^	0.630 ^a^
4	Potato_Suecia_Rodomiro_Ortiz_2023	UD	0.320 ^a^	0.634 ^a^
5	Potato_Suecia_Rodomiro_Ortiz_2023	UE	0.310 ^a^	0.634 ^a^
6	Potato_Suecia_Rodomiro_Ortiz_2023	UF	0.339 ^a^	0.632 ^a^
7	Potato_Suecia_Rodomiro_Ortiz_2023	UG	0.347 ^a^	0.630 ^a^
1	Rice_IRRI_Philippines_Spindel_2015	UA	0.037 ^b^	5.840 ^b^
2	Rice_IRRI_Philippines_Spindel_2015	UB	0.505 ^a^	0.115 ^a^
3	Rice_IRRI_Philippines_Spindel_2015	UC	0.492 ^a^	0.116 ^a^
4	Rice_IRRI_Philippines_Spindel_2015	UD	0.477 ^a^	0.118 ^a^
5	Rice_IRRI_Philippines_Spindel_2015	UE	0.485 ^a^	0.117 ^a^
6	Rice_IRRI_Philippines_Spindel_2015	UF	0.488 ^a^	0.117 ^a^
7	Rice_IRRI_Philippines_Spindel_2015	UG	0.500 ^a^	0.116 ^a^
1	Rice_Kim_2020	UA	0.258 ^c^	0.212 ^b^
2	Rice_Kim_2020	UB	0.230 ^a^	0.215 ^ab^
3	Rice_Kim_2020	UC	0.185 ^b^	0.218 ^a^
4	Rice_Kim_2020	UD	0.184 ^b^	0.218 ^a^
5	Rice_Kim_2020	UE	0.188 ^b^	0.218 ^a^
6	Rice_Kim_2020	UF	0.198 ^b^	0.217 ^a^
7	Rice_Kim_2020	UG	0.185 ^b^	0.218 ^a^
1	Summary Across Datasets	UA	0.087 ^b^	9.973 ^b^
2	Summary Across Datasets	UB	0.618 ^a^	0.287 ^a^
3	Summary Across Datasets	UC	0.612 ^a^	0.284 ^a^
4	Summary Across Datasets	UD	0.601 ^a^	0.298 ^a^
5	Summary Across Datasets	UE	0.604 ^a^	0.295 ^a^
6	Summary Across Datasets	UF	0.611 ^a^	0.282 ^a^
7	Summary Across Datasets	UG	0.614 ^a^	0.286 ^a^

## Appendix C

**Table A2:** Prediction performance for the unimodal (U) predictor of evaluated models in terms of Pearson’s correlation (COR) and Normalized Root Mean Square Error (NRMSE) for the Winter_Wheat_Washington_2020 and Winter_Wheat_Washington_2021 datasets. Tukey grouping letters summarize the descriptive ANOVA comparisons. Because repeated cross-validation observations are correlated, the letters should be interpreted together with effect magnitude and consistency across partitions, not as stand-alone confirmatory inference.

	Dataset	Methods	COR	NRMSE
1	Winter_Wheat_Washington_2020	UA	0.076 ^b^	15.383 ^b^
2	Winter_Wheat_Washington_2020	UB	0.802 ^a^	0.372 ^ac^
3	Winter_Wheat_Washington_2020	UC	0.802 ^a^	0.369 ^c^
4	Winter_Wheat_Washington_2020	UD	0.796 ^a^	0.376 ^a^
5	Winter_Wheat_Washington_2020	UE	0.798 ^a^	0.377 ^a^
6	Winter_Wheat_Washington_2020	UF	0.802 ^a^	0.367 ^c^
7	Winter_Wheat_Washington_2020	UG	0.803 ^a^	0.370 ^ac^
1	Winter_Wheat_Washington_2021	UA	0.091 ^b^	8.364 ^b^
2	Winter_Wheat_Washington_2021	UB	0.769 ^a^	0.284 ^ac^
3	Winter_Wheat_Washington_2021	UC	0.775 ^a^	0.272 ^c^
4	Winter_Wheat_Washington_2021	UD	0.765 ^a^	0.314 ^a^
5	Winter_Wheat_Washington_2021	UE	0.766 ^a^	0.305 ^ac^
6	Winter_Wheat_Washington_2021	UF	0.774 ^a^	0.266 ^c^
7	Winter_Wheat_Washington_2021	UG	0.769 ^a^	0.282 ^ac^

## Appendix D

**Table A3:** Prediction performance for the multimodal (M) predictor of evaluated models in terms of Pearson’s correlation (COR) and Normalized Root Mean Square Error (NRMSE) for the DMario_Family, Potato_Suecia, Rice_IRRI, Rice_Kim and across datasets. Tukey grouping letters summarize the descriptive ANOVA comparisons. Because repeated cross-validation observations are correlated, the letters should be interpreted together with effect magnitude and consistency across partitions, not as stand-alone confirmatory inference.

	Dataset	Method	COR	SE_COR	NRMSE
1	DMario_Family_14	MA	0.913 ^b^	0.035	0.046 ^b^
2	DMario_Family_14	MB	0.401 ^a^	0.137	1.090 ^a^
3	DMario_Family_14	MC	0.913 ^b^	0.035	0.046 ^b^
4	DMario_Family_14	MD	0.404 ^a^	0.137	1.061 ^a^
1	Potato_Suecia_Rodomiro_Ortiz_2023	MA	0.764 ^b^	0.034	0.418 ^b^
2	Potato_Suecia_Rodomiro_Ortiz_2023	MB	0.158 ^a^	0.097	9.218 ^a^
3	Potato_Suecia_Rodomiro_Ortiz_2023	MC	0.764 ^b^	0.034	0.418 ^b^
4	Potato_Suecia_Rodomiro_Ortiz_2023	MD	0.162 ^a^	0.097	9.318 ^a^
1	Rice_IRRI_Philippines_Spindel_2015	MA	0.729 ^b^	0.046	0.088 ^b^
2	Rice_IRRI_Philippines_Spindel_2015	MB	0.073 ^a^	0.066	3.613 ^a^
3	Rice_IRRI_Philippines_Spindel_2015	MC	0.728 ^b^	0.046	0.088 ^b^
4	Rice_IRRI_Philippines_Spindel_2015	MD	0.073 ^a^	0.062	3.655 ^a^
1	Rice_Kim_2020	MA	0.949 ^a^	0.001	0.070 ^a^
2	Rice_Kim_2020	MB	0.945 ^a^	0.001	0.072 ^a^
3	Rice_Kim_2020	MC	0.938 ^a^	0.001	0.076 ^a^
4	Rice_Kim_2020	MD	0.955 ^a^	0.001	0.065 ^b^
1	Summary_Across	MA	0.841 ^b^	0.044	0.191 ^b^
2	Summary_Across	MB	0.145 ^a^	0.089	5.044 ^a^
3	Summary_Across	MC	0.842 ^b^	0.043	0.190 ^b^
4	Summary_Across	MD	0.143 ^a^	0.089	5.012 ^a^

## Appendix E

**Table A4:** Prediction performance for the multimodal (M) predictor of evaluated models in terms of Pearson’s correlation (COR) and Normalized Root Mean Square Error (NRMSE) for the Winter_Wheat_Washington_2020 and Winter_Wheat_Washington_2021 datasets. Tukey grouping letters summarize the descriptive ANOVA comparisons. Because repeated cross-validation observations are correlated, the letters should be interpreted together with effect magnitude and consistency across partitions, not as stand-alone confirmatory inference.

	Dataset	Method	COR	NRMSE
1	Winter_Wheat_Washington_2020	MA	0.891 ^b^	0.296 ^b^
2	Winter_Wheat_Washington_2020	MB	0.122 ^a^	7.437 ^a^
3	Winter_Wheat_Washington_2020	MC	0.894 ^b^	0.292 ^b^
4	Winter_Wheat_Washington_2020	MD	0.117 ^a^	7.249 ^a^
1	Winter_Wheat_Washington_2021	MA	0.952 ^b^	0.137 ^b^
2	Winter_Wheat_Washington_2021	MB	0.143 ^a^	3.487 ^a^
3	Winter_Wheat_Washington_2021	MC	0.952 ^b^	0.137 ^b^
4	Winter_Wheat_Washington_2021	MD	0.135 ^a^	3.469 ^a^

## Appendix F

**Table A5:** Variance components and heritability for models UG and MA for each dataset and trait. The underscore (_) indicates that the corresponding component was not estimated because environmental and G × E variance components were included only in the multimodal model. Numerically near-zero variance estimates were treated as zero for heritability summaries.

Dataset	Trait	Model	$\sigma_{E}^{2}$	$\sigma_{g}^{2}$	$\sigma_{gE}^{2}$	$\sigma_{e}^{2}$	H
DMario_Family_14	GM_Trial	M	0.002	159.176	0.009	0.011	1.000
DMario_Family_14	GM_Trial	U	_	159.199	_	0.008	1.000
DMario_Family_14	BLUE_Yield_corrected	M	300,322.034	35,861.059	_	_	0.101
DMario_Family_14	BLUE_Yield_corrected	U	_	49,129.462	_	_	0.566
DMario_Family_14	BLUE_Moisture	M	2.406	0.074	0.133	0.163	0.029
DMario_Family_14	BLUE_Moisture	U	_	0.096	_	3.654	0.240
Potato_Suecia_Rodomiro_Ortiz_2023	BLUE_40mm	M	0.159	0.209	0.050	0.202	0.510
Potato_Suecia_Rodomiro_Ortiz_2023	BLUE_40mm	U	_	0.139	_	0.519	0.615
Potato_Suecia_Rodomiro_Ortiz_2023	BLUE_40.50mm	M	0.180	0.328	0.104	0.777	0.501
Potato_Suecia_Rodomiro_Ortiz_2023	BLUE_40.50mm	U	_	0.267	_	1.173	0.577
Potato_Suecia_Rodomiro_Ortiz_2023	BLUE_50.60mm	M	0.507	0.487	0.217	1.539	0.378
Potato_Suecia_Rodomiro_Ortiz_2023	BLUE_50.60mm	U	_	0.332	_	2.605	0.434
Potato_Suecia_Rodomiro_Ortiz_2023	BLUE_60mm	M	4.233	3.173	0.776	3.752	0.389
Potato_Suecia_Rodomiro_Ortiz_2023	BLUE_60mm	U	_	1.663	_	11.302	0.469
Potato_Suecia_Rodomiro_Ortiz_2023	BLUE_totalfractionedweight	M	5.104	5.038	0.948	5.137	0.452
Potato_Suecia_Rodomiro_Ortiz_2023	BLUE_totalfractionedweight	U	_	3.044	_	14.434	0.559
Potato_Suecia_Rodomiro_Ortiz_2023	BLUE_starch	M	9.396	3.365	0.500	3.021	0.252
Potato_Suecia_Rodomiro_Ortiz_2023	BLUE_starch	U	_	0.879	_	19.260	0.215
Potato_Suecia_Rodomiro_Ortiz_2023	BLUE_sugar	M	0.500	0.325	0.086	0.446	0.356
Potato_Suecia_Rodomiro_Ortiz_2023	BLUE_sugar	U	_	0.170	_	1.301	0.440
Rice_IRRI_Philippines_Spindel_2015	PH	M	21.324	43.605	6.282	11.868	0.589
Rice_IRRI_Philippines_Spindel_2015	PH	U	_	20.152	_	81.462	0.331
Rice_IRRI_Philippines_Spindel_2015	FLW	M	9.905	16.103	3.031	4.144	0.544
Rice_IRRI_Philippines_Spindel_2015	FLW	U	_	6.802	_	29.664	0.314
Rice_IRRI_Philippines_Spindel_2015	Lg	M	0.006	0.319	0.115	0.370	0.562
Rice_IRRI_Philippines_Spindel_2015	Lg	U	_	0.318	_	0.473	0.573
Rice_IRRI_Philippines_Spindel_2015	Exs	M	0.005	0.002	0.005	0.009	0.168
Rice_IRRI_Philippines_Spindel_2015	Exs	U	_	0.004	_	0.024	0.239
Rice_IRRI_Philippines_Spindel_2015	PnN	M	0.001	0.069	0.071	0.356	0.244
Rice_IRRI_Philippines_Spindel_2015	PnN	U	_	0.115	_	0.382	0.376
Rice_IRRI_Philippines_Spindel_2015	PedL	M	0.128	1.139	0.157	0.668	0.678
Rice_IRRI_Philippines_Spindel_2015	PedL	U	_	0.810	_	1.205	0.573
Rice_IRRI_Philippines_Spindel_2015	PnL	M	0.081	1.473	0.130	0.492	0.790
Rice_IRRI_Philippines_Spindel_2015	PnL	U	_	1.067	_	0.881	0.708
Rice_IRRI_Philippines_Spindel_2015	Flg.LL	M	0.591	3.208	0.676	1.851	0.634
Rice_IRRI_Philippines_Spindel_2015	Flg.LL	U	_	1.972	_	4.338	0.476
Rice_IRRI_Philippines_Spindel_2015	Flg.LW	M	0.000	0.004	0.002	0.005	0.565
Rice_IRRI_Philippines_Spindel_2015	Flg.LW	U	_	0.005	_	0.006	0.621
Rice_IRRI_Philippines_Spindel_2015	Flg.LA	M	0.250	3.319	1.237	2.803	0.594
Rice_IRRI_Philippines_Spindel_2015	Flg.LA	U	_	3.063	_	4.488	0.577
Rice_IRRI_Philippines_Spindel_2015	SPn	M	99.304	20.577	18.394	40.127	0.138
Rice_IRRI_Philippines_Spindel_2015	SPn	U	_	24.692	_	284.437	0.148
Rice_IRRI_Philippines_Spindel_2015	FGP	M	978.217	1145.383	1065.818	4176.787	0.241
Rice_IRRI_Philippines_Spindel_2015	FGP	U	_	1033.021	_	7175.402	0.224
Rice_IRRI_Philippines_Spindel_2015	GrL	M	0.006	0.146	0.014	0.040	0.818
Rice_IRRI_Philippines_Spindel_2015	GrL	U	_	0.119	_	0.071	0.771
Rice_IRRI_Philippines_Spindel_2015	GrW	M	0.001	0.007	0.002	0.005	0.596
Rice_IRRI_Philippines_Spindel_2015	GrW	U	_	0.005	_	0.010	0.530
Rice_IRRI_Philippines_Spindel_2015	LBR	M	0.001	0.068	0.006	0.021	0.820
Rice_IRRI_Philippines_Spindel_2015	LBR	U	_	0.067	_	0.028	0.829
Rice_IRRI_Philippines_Spindel_2015	X1000GW	M	0.044	2.297	0.223	0.907	0.791
Rice_IRRI_Philippines_Spindel_2015	X1000GW	U	_	2.124	_	1.193	0.781
Rice_IRRI_Philippines_Spindel_2015	YPP	M	4.365	1.129	0.964	2.740	0.154
Rice_IRRI_Philippines_Spindel_2015	YPP	U	_	0.993	_	9.953	0.166
Rice_IRRI_Philippines_Spindel_2015	YLD	M	911.532	_	_	_	0.647
Rice_IRRI_Philippines_Spindel_2015	YLD	U	_	_	_	_	0.679
Rice_Kim_2020	y.pheno	M	3277.706	85,206.945	_	61,969.671	0.810
Rice_Kim_2020	y.pheno	U	_	13,734.911	_	_	0.204
Winter_Wheat_Washington_2020	GY	M	1131.723	42.409	43.864	97.196	0.035
Winter_Wheat_Washington_2020	GY	U	_	539.286	_	765.527	0.809
Winter_Wheat_Washington_2020	PH	M	521.543	151.419	79.354	55.776	0.218
Winter_Wheat_Washington_2020	PH	U	_	311.065	_	208.715	0.899
Winter_Wheat_Washington_2020	HD	M	899.028	142.860	237.650	91.598	0.130
Winter_Wheat_Washington_2020	HD	U	_	293.330	_	742.129	0.703
Winter_Wheat_Washington_2020	Can_Cover	M	678.042	39.699	35.173	86.737	0.054
Winter_Wheat_Washington_2020	Can_Cover	U	_	264.750	_	393.608	0.801
Winter_Wheat_Washington_2020	NM_900	M	43.546	2118.302	31.563	69.407	0.972
Winter_Wheat_Washington_2020	NM_900	U	_	2596.929	_	80.018	0.995
Winter_Wheat_Washington_2020	NM_975	M	88.990	564.437	38.624	73.577	0.840
Winter_Wheat_Washington_2020	NM_975	U	_	913.222	_	114.599	0.980
Winter_Wheat_Washington_2020	Blue	M	41.607	9.734	5.870	9.281	0.181
Winter_Wheat_Washington_2020	Blue	U	_	28.985	_	29.356	0.856
Winter_Wheat_Washington_2020	Green	M	281.314	58.345	29.501	35.112	0.166
Winter_Wheat_Washington_2020	Green	U	_	110.199	_	208.202	0.761
Winter_Wheat_Washington_2020	NDRE1	M	0.001	0.003	0.000	0.000	0.749
Winter_Wheat_Washington_2020	NDRE1	U	_	0.002	_	0.001	0.909
Winter_Wheat_Washington_2020	NDVI	M	0.000	0.061	0.001	0.002	0.990
Winter_Wheat_Washington_2020	NDVI	U	_	0.065	_	0.002	0.996
Winter_Wheat_Washington_2020	NIR	M	3.443	2532.235	25.494	58.534	0.993
Winter_Wheat_Washington_2020	NIR	U	_	2985.716	_	33.946	0.998
Winter_Wheat_Washington_2020	RE1	M	947.871	120.847	82.928	85.792	0.110
Winter_Wheat_Washington_2020	RE1	U	_	371.838	_	627.431	0.781
Winter_Wheat_Washington_2020	RE2	M	2.240	1779.433	16.987	39.025	0.994
Winter_Wheat_Washington_2020	RE2	U	_	2063.539	_	23.487	0.998
Winter_Wheat_Washington_2020	Red	M	511.863	169.969	103.366	76.131	0.239
Winter_Wheat_Washington_2020	Red	U	_	253.094	_	404.170	0.790
Winter_Wheat_Washington_2020	MTVI	M	0.001	0.000	0.000	0.000	0.032
Winter_Wheat_Washington_2020	MTVI	U	_	0.001	_	0.000	0.979
Winter_Wheat_Washington_2020	NWI1	M	0.000	0.000	0.000	0.000	0.044
Winter_Wheat_Washington_2020	NWI1	U	_	0.000	_	0.000	0.980
Winter_Wheat_Washington_2021	GY	M	486.781	69.209	27.788	53.206	0.122
Winter_Wheat_Washington_2021	GY	U	_	281.884	_	341.245	0.869
Winter_Wheat_Washington_2021	PH	M	359.349	31.214	24.638	36.364	0.078
Winter_Wheat_Washington_2021	PH	U	_	263.330	_	237.454	0.899
Winter_Wheat_Washington_2021	HD	M	1084.114	55.537	111.640	61.919	0.048
Winter_Wheat_Washington_2021	HD	U	_	1014.201	_	218.569	0.974
Winter_Wheat_Washington_2021	Can_Cover	M	275.313	36.257	11.463	46.316	0.114
Winter_Wheat_Washington_2021	Can_Cover	U	_	43.613	_	214.530	0.619
Winter_Wheat_Washington_2021	NM_900	M	199.084	35.011	23.318	32.555	0.145
Winter_Wheat_Washington_2021	NM_900	U	_	474.980	_	48.540	0.987
Winter_Wheat_Washington_2021	NM_975	M	105.307	25.464	21.509	25.555	0.186
Winter_Wheat_Washington_2021	NM_975	U	_	163.673	_	68.537	0.950
Winter_Wheat_Washington_2021	Blue	M	38.107	4.887	3.057	7.171	0.110
Winter_Wheat_Washington_2021	Blue	U	_	12.538	_	45.427	0.688
Winter_Wheat_Washington_2021	Green	M	78.767	13.985	4.596	13.373	0.147
Winter_Wheat_Washington_2021	Green	U	_	28.542	_	74.695	0.754
Winter_Wheat_Washington_2021	NDRE1	M	0.000	0.000	0.000	0.000	0.184
Winter_Wheat_Washington_2021	NDRE1	U	_	0.001	_	0.000	0.935
Winter_Wheat_Washington_2021	NDVI	M	0.012	0.000	0.000	0.000	0.021
Winter_Wheat_Washington_2021	NDVI	U	_	0.027	_	0.002	0.991
Winter_Wheat_Washington_2021	NIR	M	208.790	37.668	30.437	26.647	0.149
Winter_Wheat_Washington_2021	NIR	U	_	739.970	_	8.649	0.999
Winter_Wheat_Washington_2021	RE1	M	201.866	31.231	9.939	26.446	0.131
Winter_Wheat_Washington_2021	RE1	U	_	51.983	_	158.089	0.725
Winter_Wheat_Washington_2021	RE2	M	131.472	40.443	29.468	22.031	0.227
Winter_Wheat_Washington_2021	RE2	U	_	584.323	_	7.118	0.998
Winter_Wheat_Washington_2021	Red	M	197.978	19.266	7.401	18.706	0.087
Winter_Wheat_Washington_2021	Red	U	_	83.443	_	150.345	0.816
Winter_Wheat_Washington_2021	MTVI	M	0.001	0.000	0.000	0.000	0.065
Winter_Wheat_Washington_2021	MTVI	U	_	0.001	_	0.001	0.924
Winter_Wheat_Washington_2021	NWI1	M	0.001	0.000	0.000	0.000	0.021
Winter_Wheat_Washington_2021	NWI1	U	_	0.001	_	0.001	0.927

## Appendix G

**Table A6:** Sensitivity analysis for the nine combinations of hyperparameters (CH) for the dataset Rice_Kim_2020 for the unimodal predictor of model UG. Mean and SD denote the mean and standard deviation of the performance under each metric. COR denotes the Pearson’s correlation and NRMSE denotes the Normalized Root Mean Square Error.

CH	Metric	Mean	SD
CH_1	COR	0.181	0.021
CH_1	NRMSE	0.220	0.003
CH_2	COR	0.177	0.022
CH_2	NRMSE	0.218	0.004
CH_3	COR	0.186	0.026
CH_3	NRMSE	0.218	0.005
CH_4	COR	0.168	0.020
CH_4	NRMSE	0.219	0.003
CH_5	COR	0.184	0.030
CH_5	NRMSE	0.218	0.003
CH_6	COR	0.195	0.016
CH_6	NRMSE	0.216	0.002
CH_7	COR	0.161	0.022
CH_7	NRMSE	0.218	0.005
CH_8	COR	0.187	0.021
CH_8	NRMSE	0.218	0.005
CH_9	COR	0.189	0.024
CH_9	NRMSE	0.215	0.005

**Table A7:** Sensitivity analysis for the nine combinations of hyperparameters (CH) for the dataset Rice_Kim_2020 for the multimodal predictor of model MA. Mean and SD denote the mean and standard deviation of the performance under each metric. COR denotes the Pearson’s correlation and NRMSE denotes the Normalized Root Mean Square Error.

CH	Metric	Mean	SD
CH_1	COR	0.947	0.004
CH_1	NRMSE	0.071	0.003
CH_2	COR	0.950	0.003
CH_2	NRMSE	0.070	0.001
CH_3	COR	0.954	0.002
CH_3	NRMSE	0.067	0.003
CH_4	COR	0.944	0.006
CH_4	NRMSE	0.073	0.004
CH_5	COR	0.948	0.003
CH_5	NRMSE	0.071	0.002
CH_6	COR	0.954	0.003
CH_6	NRMSE	0.068	0.002
CH_7	COR	0.935	0.008
CH_7	NRMSE	0.078	0.004
CH_8	COR	0.944	0.003
CH_8	NRMSE	0.074	0.002
CH_9	COR	0.952	0.003
CH_9	NRMSE	0.068	0.002
